# The CLCA1/TMEM16A/Cl^–^ current axis associates with H_2_S deficiency in diabetic kidney injury

**DOI:** 10.1172/jci.insight.174848

**Published:** 2025-01-09

**Authors:** Hak Joo Lee, Yuyang Sun, Falguni Das, Wenjun Ju, Viji Nair, Christopher G. Kevil, Shankara Varadarajan, Guanshi Zhang, Goutam Ghosh Choudhury, Brij B. Singh, Matthias Kretzler, Robert G. Nelson, Kumar Sharma, Balakuntalam S. Kasinath

**Affiliations:** 1 Center for Precision Medicine, Department of Medicine, and; 2 Department of Periodontics, University of Texas Health, San Antonio, San Antonio, Texas, USA.; 3 Department of Internal Medicine and; 4 Department of Computational Medicine and Bioinformatics, University of Michigan Medical School, Ann Arbor, Michigan, USA.; 5 Department of Pathology, Louisiana State University Health Science Center, Shreveport, Louisiana, USA.; 6 Research service and; 7 Geriatric Research Education and Clinical Center, South Texas Veterans Health Care System, San Antonio, Texas, USA.; 8 National Institute of Diabetes and Digestive and Kidney Diseases, Phoenix, Arizona, USA.; 9 Joslin Diabetes Center, Boston, Massachusetts, USA.

**Keywords:** Nephrology, Therapeutics, Chloride channels, Diabetes, Extracellular matrix

## Abstract

The role played by anionic channels in diabetic kidney disease (DKD) is not known. Chloride channel accessory 1 (CLCA1) facilitates the activity of TMEM16A (Anoctamin-1), a Ca^2+^-dependent Cl^–^ channel. We examined if CLCA1/TMEM16A had a role in DKD. In mice with type 2 diabetes, renal cortical CLCA1 and TMEM16A content was increased. CLCA1 and TMEM16A content was associated with hydrogen sulfide (H_2_S) deficiency, mTOR complex 1 (mTORC1) activation, albuminuria, and matrix increase. Administering sodium hydrosulfide (NaHS), a source of H_2_S, mitigated these changes. In proximal tubular epithelial (MCT) cells, high glucose rapidly increased CLCA1 by recruiting the IL-6/STAT3 axis and augmented TMEM16A expression by stimulating its mRNA translation; these changes were abolished by NaHS. Patch clamp experiments showed that high glucose increased Cl^–^ current in MCT cells that was ameliorated by NaHS and a TMEM16A chemical inhibitor. siRNA against CLCA1 or TMEM16A and TMEM16A inhibitor abolished high glucose–induced mTORC1 activation and matrix protein increase. Tubular expression of *TMEM16A* correlated with albuminuria in kidney biopsies from people with type 2 diabetes. We report a pathway for DKD in which H_2_S deficiency results in kidney injury by the recruitment of the CLCA1/TMEM16A/Cl^–^ current system.

## Abstract 2

Dysfunction of CLCA1-TMEM16A-chloride current axis associates with H2S deficiency and contributes to mTOR signaling, albuminuria and matrix increase in diabetic kidney injury.

## Graphical abstract

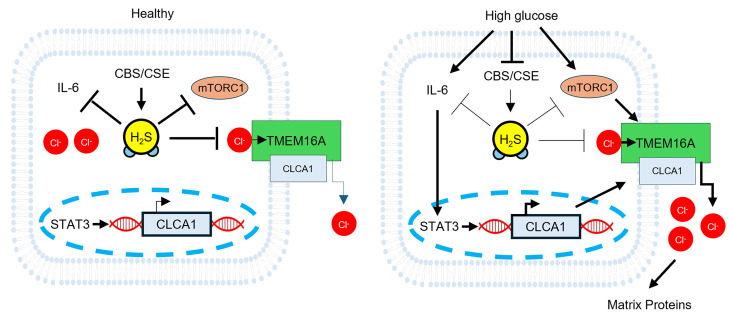

## Introduction

Unabated tubulointerstitial fibrosis is a major determinant of progressive loss of kidney function culminating in end-stage kidney disease (1). Tubulointerstitial fibrosis is a strong predictor of loss of kidney function in diabetes (2, 3) and chronic kidney disease of diverse etiology (1). Diabetes is the most common cause of end-stage kidney disease in the United States (4). At present the management of diabetic kidney disease (DKD) leaves much to be desired. Although we can slow the progression to end-stage kidney disease, we have not developed strategies that arrest DKD. New approaches have become necessary.

Recently, we adopted an RNA-Seq strategy as a discovery approach to explore aging-induced kidney fibrosis in mice and found an association with increase in *Clca1* mRNA in the kidney (5). CLCA1 is an accessory protein that combines with and promotes the activity of TMEM16A (Anoctamin-1), a Ca^2+^-dependent Cl^–^ channel located in the plasma membrane (6, 7). CLCA1 functions as a self-cleaving protease; the N-terminal fragment translocates to bind TMEM16A and augment Cl^–^ current (8). CLCA1 does not function as a Cl^–^ channel on its own.

Several Cl^–^ channels are expressed in the kidney, including the CLC gene family, cystic fibrosis transmembrane conductance regulator, volume-regulated channels, ligand-gated channels, maxi-ion channels, and Ca^2+^-dependent Cl^–^ channels (6, 7). TMEM16A belongs to the last family, which has 10 members (TMEM16A–K) (9–11). TMEM16A has 10 transmembrane domains with intracellular N- and C-terminals (12). TMEM16A is expressed by podocytes and cortical proximal and distal tubule epithelial cells (13, 14). It is involved in Cl^–^ secretion, albumin uptake, and endosomal acidification (13). Targeted deletion of TMEM16A in the podocyte does not seem to affect glomerular filtration or increase urinary albumin loss; however, loss of TMEM16A in the tubules causes transient proteinuria (13). Combined deletion of TMEM16A in podocyte and tubules leads to nephropenia, glomerular enlargement, tubular injury, and proteinuria (15). TMEM16A-knockout mice die prematurely because of structural abnormalities of the respiratory tract (16, 17).

There is a growing awareness that the CLCA1/TMEM16A system is involved in kidney pathology. Cl^–^ secretion by TMEM16A contributes to cyst growth in polycystic kidney disease (14, 18–20). TMEM16A plays a role in hypertension (21). Aging-associated kidney injury correlates with increased expression of CLCA1 (5). Renal fibrosis following high-fat diet administration and unilateral ureteral obstruction is associated with increased TMEM16A expression (22).

It is unknown whether CLCA1/TMEM16A is involved in diabetic kidney injury. The role of ion channels is not well studied in DKD. Given that ion channels extensively regulate electrolyte and proton transport across the membranes of tubular and glomerular cells, this lack of information on their role in DKD is even more striking. While some knowledge exists on the role of cationic channels in DKD, there is a paucity of information on anionic channels, particularly chloride channels, including the TMEM16A.

Gasotransmitters including nitric oxide, carbon monoxide, and hydrogen sulfide (H_2_S) are gaining increasing attention for their regulatory role in kidney health and disease, including diabetes (23, 24). In nearly all the kidney cells, H_2_S is constitutively synthesized by cystathionine β-synthase (CBS) and cystathionine γ-lyase (CSE) in the cytosolic trans-sulfuration pathway with additional contributions from other cell compartments. RNA-Seq studies have confirmed variable expression of CBS and CSE in tubular segments along the nephron, including the proximal tubules (25). Kidney CBS and CSE expression is reduced in diabetic and aging rodents (26–29). Our recent investigations revealed that the CLCA1/TMEM16A/Cl^–^ current system is involved in kidney injury in aging mice and that H_2_S is an upstream regulator of this axis in the aging kidney (5). Lack of information on the CLCA1/TMEM16A/Cl^–^ current system and its relation to H_2_S in DKD prompted us to pursue the current study.

## Results

### Renal cortical expression of CLCA1 and TMEM16A is increased in diabetes.

**Figure 1:**
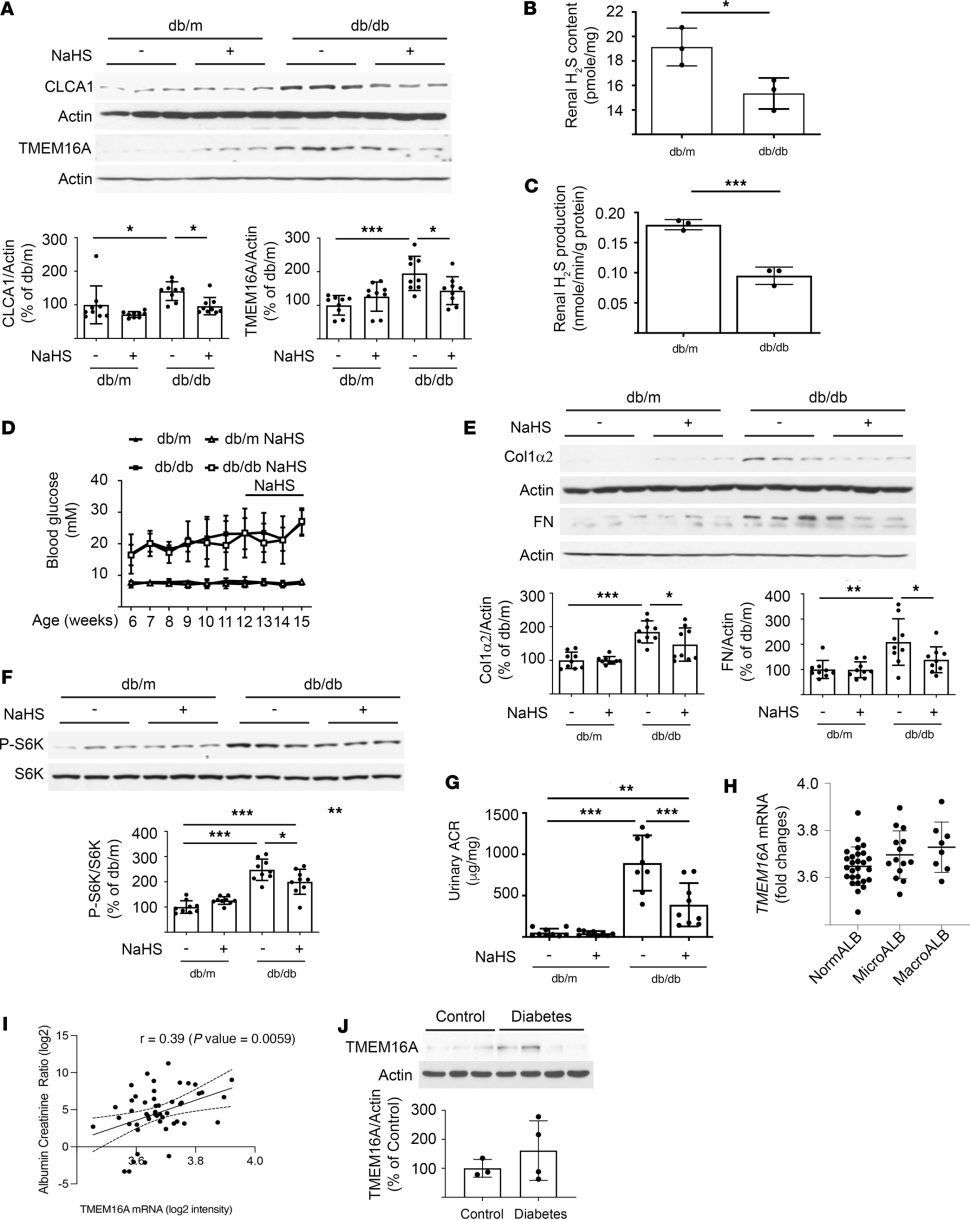
Regulation of renal cortical CLCA1 and TMEM16A in mice with type 2 diabetes. (**A**) Renal cortical expression of CLCA1 and TMEM16A was increased in diabetic mice compared with nondiabetic controls, and it was inhibited by the administration of NaHS for 3 weeks. (**B**) Renal cortical H_2_S content was decreased in diabetic mice. (**C**) H_2_S generation was decreased in the kidney cortex of db/db diabetic mice by 45% compared with db/m control mice (0.10 ± 0.01 vs. 0.18 ± 0.01 nmol/g/min, mean ± SD, respectively, *P* < 0.001). (**D**) Blood glucose levels were not affected by NaHS. (**E**) Diabetes-induced increase in renal cortical content of collagen 1α2 and fibronectin was inhibited by NaHS. (**F**) Phosphorylation of p70 S6 kinase (P-S6K) was increased in the renal cortex of diabetic mice, indicating mTORC1 activation; it was inhibited by NaHS. (**G**) Diabetes-induced increase in urinary albumin-to-creatinine ratio (urinary ACR) was reduced by NaHS. Data (mean ± SD) from 9 mice in each group of mice (db/m nondiabetic controls treated with or without NaHS, db/db diabetic mice treated with or without NaHS) are shown in bars with scatterplots and were analyzed (**A** and **D**–**F**) by ANOVA. Data from 3 mice in each group are presented (**B** and **C**) and were analyzed by *t* test. *P < 0.05, **P < 0.01, ***P < 0.001. (**H**) Renal tubular TMEM16A expression significantly correlated with the degree of albuminuria in 49 American Indian individuals with diabetes (*P* = 0.0059 by ANOVA). NormALB, normo-albuminuria; microALB, micro-albuminuria; and macroALB, macroalbuminuria. (**I**) There was a significant correlation between TMEM16A mRNA content in the kidney tubulointerstitium and urinary albumin-to-creatinine ratio by Pearson’s correlation coefficient. (**J**) TMEM16A expression in the human kidneys of control (*n* = 3) and diabetes individuals (*n* = 4).

Kidney cortical expression of both CLCA1 and TMEM16A was increased in type 2 diabetic mice (Figure 1A). This was associated with reduced H_2_S content and generation in the kidney cortex in diabetic mice (Figure 1, B and C). In combination with our previous report that renal cortical expression of CBS and CSE is reduced in diabetic mice (27), these data demonstrate that decreased enzymatic generation leads to reduced kidney H_2_S content in diabetes. mTOR complex 1 (mTORC1) activation mediates diabetes-induced kidney matrix protein increase and albuminuria in rodents (30–32). Renal cortical content of extracellular matrix proteins collagen 1α2 and fibronectin was increased in diabetes (Figure 1E). Increased Thr389 phosphorylation of p70 S6 kinase indicated that renal cortical mTORC1 was activated in diabetic mice (Figure 1F). Urinary albumin excretion was increased in diabetic mice (Figure 1G).

To explore the role of H_2_S deficiency in kidney injury in diabetic mice, we randomized control db/m and diabetic db/db mice to receive water alone or sodium hydrosulfide (NaHS) administered in drinking water for 3 weeks. NaHS did not affect blood glucose levels in nondiabetic or diabetic mice (Figure 1D). Increase in renal cortical CLCA1 and TMEM16A and matrix protein content in diabetic mice was abrogated by NaHS (Figure 1, A and E), as were mTORC1 activation and albuminuria (Figure 1, F and G).

### CLCA1 and TMEM16A expression in people with DKD.

We analyzed the relationship between tubular expression of *CLCA1* and *TMEM16A* in early-stage DKD in American Indians. Clinical data on the participants are shown in Table 1 (*n* = 49). Of the participants with diabetes, 27 had normal urinary albumin-to-creatinine ratio (stage A1), 14 had microalbuminuria (stage A2), and 8 had macroalbuminuria (stage A3). Among participants with diabetes there was a positive correlation between tubular *TMEM16A* expression (but not *CLCA1*) and urinary albumin-to-creatinine ratio (*r* value 0.39, *P* = 0.0059; Figure 1, H and I). Immunoblotting showed a trend toward increase in TMEM16A protein expression in the kidney cortex of participants with diabetes (non-American Indians) compared with nondiabetic controls (Figure 1J).

### High glucose increases CLCA1 via the IL-6/STAT3 axis.

**Figure 2:**
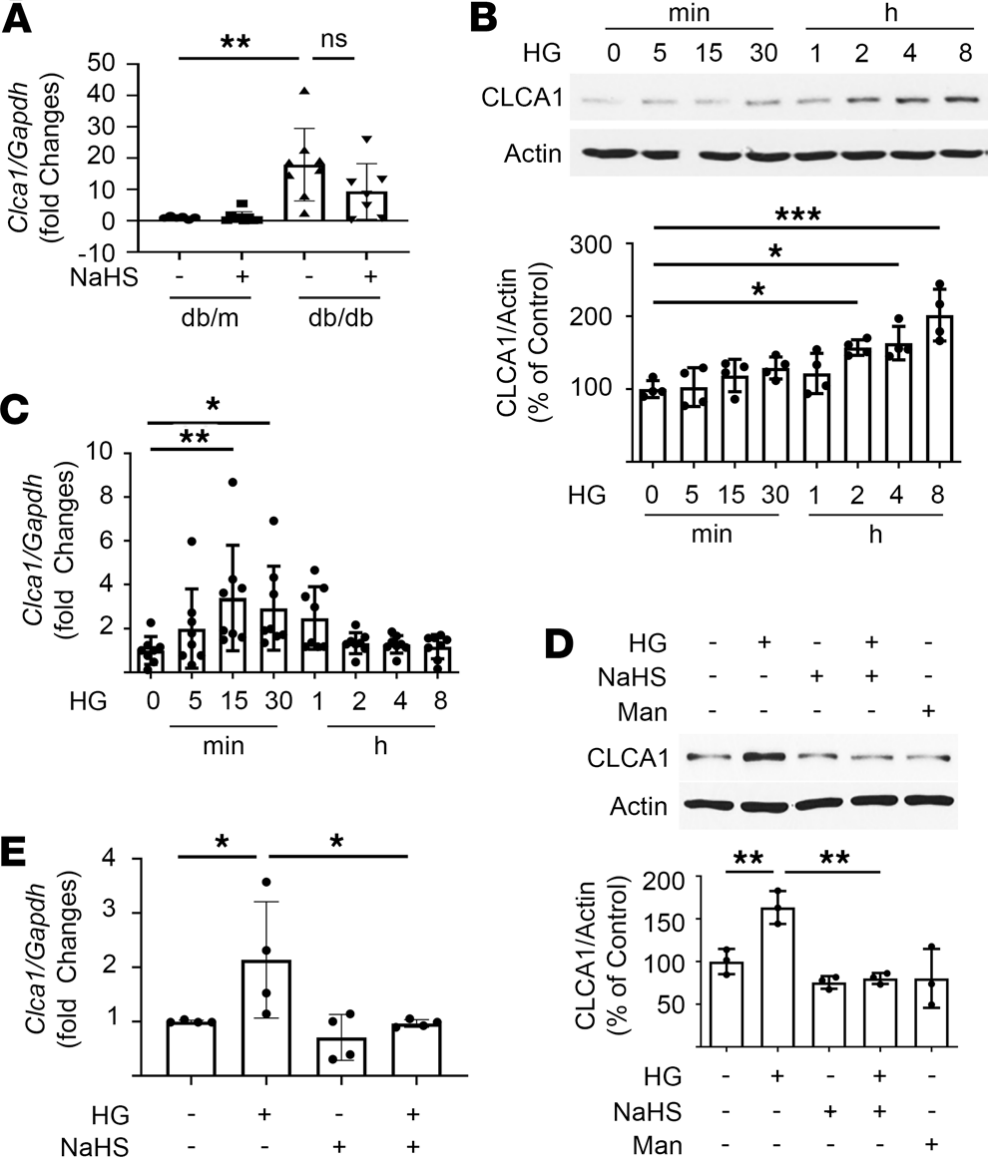
High glucose regulation of CLCA1. (**A**) Quantitative reverse transcription PCR (qRT-PCR) showed that *Clca1* mRNA was increased in the renal cortical lysates from diabetic mice; NaHS treatment showed a trend toward abrogation of *Clca1* mRNA increase (*n* = 6–9/group, **P < 0.01 vs. db/m mice). (**B**) High glucose increased CLCA1 expression in proximal tubular epithelial cells in a time-dependent manner as shown by immunoblotting. (**C**) qRT-PCR showed rapid increase in *Clca1* mRNA following exposure to high glucose. (**D**) Immunoblotting showed that NaHS inhibited high glucose–induced increase in CLCA1; equimolar mannitol did not affect CLCA1 expression. (**E**) NaHS abrogated increase in *Clca1* mRNA induced by high glucose. (**B**–**E**) Data from 4–8 experiments (mean ± SD) are shown in bars with scatterplots and were analyzed by ANOVA. *P < 0.05, **P < 0.01, ***P < 0.001.

Renal cortical content of *Clca1* mRNA was increased in diabetic mice, suggesting that increased transcription drives protein expression; NaHS treatment showed a trend toward abrogation of *Clca1* mRNA increase (Figure 2A). Proximal tubule epithelial cells express CLCA1 in the kidney (5, 25). Accordingly, we examined mechanisms underlying glucose regulation of CLCA1 in vitro in MCT cells. High glucose time-dependently increased CLCA1 protein expression (Figure 2B) and *Clca1* mRNA, the latter preceding the increase in protein (Figure 2C). NaHS inhibited high glucose–induced increase in CLCA1 protein and *Clca1* mRNA (Figure 2, D and E), corresponding to in vivo observations (Figure 1A). Osmotic control mannitol did not affect CLCA1 protein expression in MCT cells (Figure 2D).

**Figure 3:**
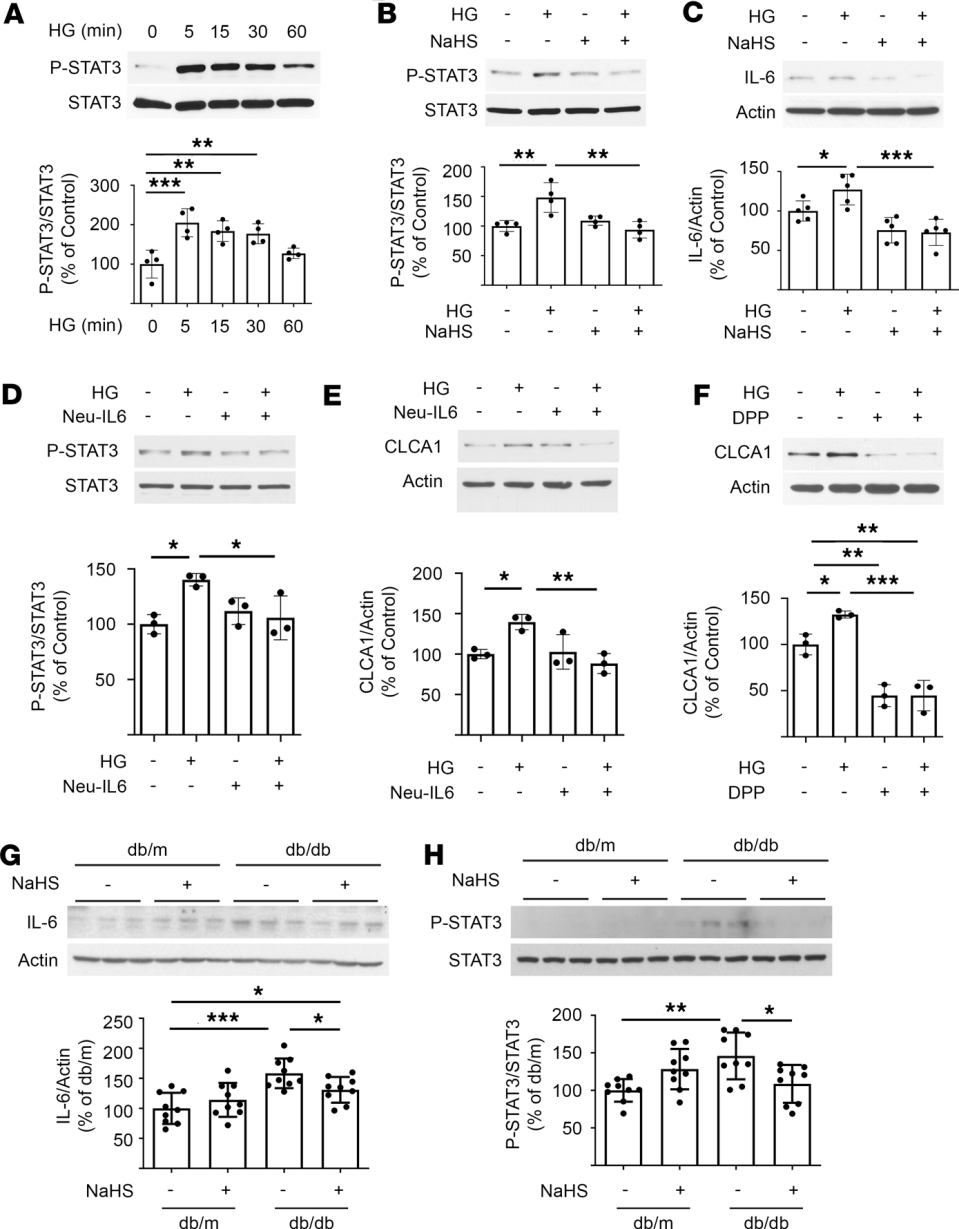
IL-6 and STAT3 mediate high glucose induction of CLCA1 in diabetic kidney injury. (**A**) High glucose rapidly induced STAT3 phosphorylation with onset at 5 minutes. (**B**) High glucose–induced STAT3 phosphorylation was inhibited by NaHS. (**C**) High glucose increased IL-6 expression within 5 minutes, which was inhibited by NaHS. (**D** and **E**) Murine proximal tubular epithelial (MCT) cells were preincubated with 2 mg/mL of IL-6 neutralizing antibody for 30 minutes, and then cells were incubated with 30 mM glucose for 5 minutes (STAT3) or 2 hours (CLCA1). (**F**) Cells were preincubated with 10 μM 5,15-DPP (a STAT3 inhibitor) for 30 minutes followed by incubation with 30 mM glucose for 2 hours. (**G** and **H**) Expression of IL-6 and phosphorylated STAT3 was increased in the renal cortex of diabetic mice that was inhibited by NaHS. Data from 4–5 experiments (mean ± SD) are shown in bars with scatterplots and were analyzed by ANOVA. Data from 9 mice in each group (mean ± SD) are shown in bars with scatterplots and were analyzed by ANOVA. *P < 0.05, **P < 0.01, ***P < 0.001.

STAT3, a transcription factor, stimulates *Clca1* mRNA expression in mouse mammary glands (33). STAT3 is activated by phosphorylation at Tyr705, which allows it to homodimerize or heterodimerize with other STATs, translocate to the nucleus, and regulate transcription. High glucose induced STAT3 phosphorylation within 5 minutes (Figure 3A) preceding increase in *Clca1* mRNA. Interestingly, this was inhibited by coincubation with NaHS (Figure 3B). IL-6 is known to promote STAT3 phosphorylation (34). High glucose increased IL-6 expression at 5 minutes, which was inhibited by NaHS (Figure 3C).

We examined the requirement of IL-6 for high glucose regulation of CLCA1 expression. Neutralizing antibody against IL-6 inhibited high glucose–induced increase in STAT3 phosphorylation and CLCA1 expression (Figure 3, D and E), indicating that STAT3 activation is IL-6 dependent and that IL-6 is required for high glucose stimulation of CLCA1 expression. As STAT3 mediates IL-6’s effects, we explored the role of STAT3 in mediating high glucose’s effect on CLCA1 expression. A selective STAT3 inhibitor, 5,15-diphenyl-21H,23H-porphine (DPP), abrogated high glucose–induced increase in CLCA1 expression (Figure 3F). These data establish the requirement of IL-6 signaling via STAT3 for high glucose–induced increase in CLCA1 expression. We evaluated the status of IL-6 and STAT3 in the kidney cortex of diabetic mice for in vivo relevance of these findings. Diabetes increased renal cortical expression of IL-6 and phosphorylated STAT3, which was inhibited by NaHS (Figure 3, G and H). These data suggest that the IL-6/STAT3 axis stimulates *Clca1* transcription in DKD.

### CLCA1 mediates high glucose induction of matrix protein expression.

**Figure 4:**
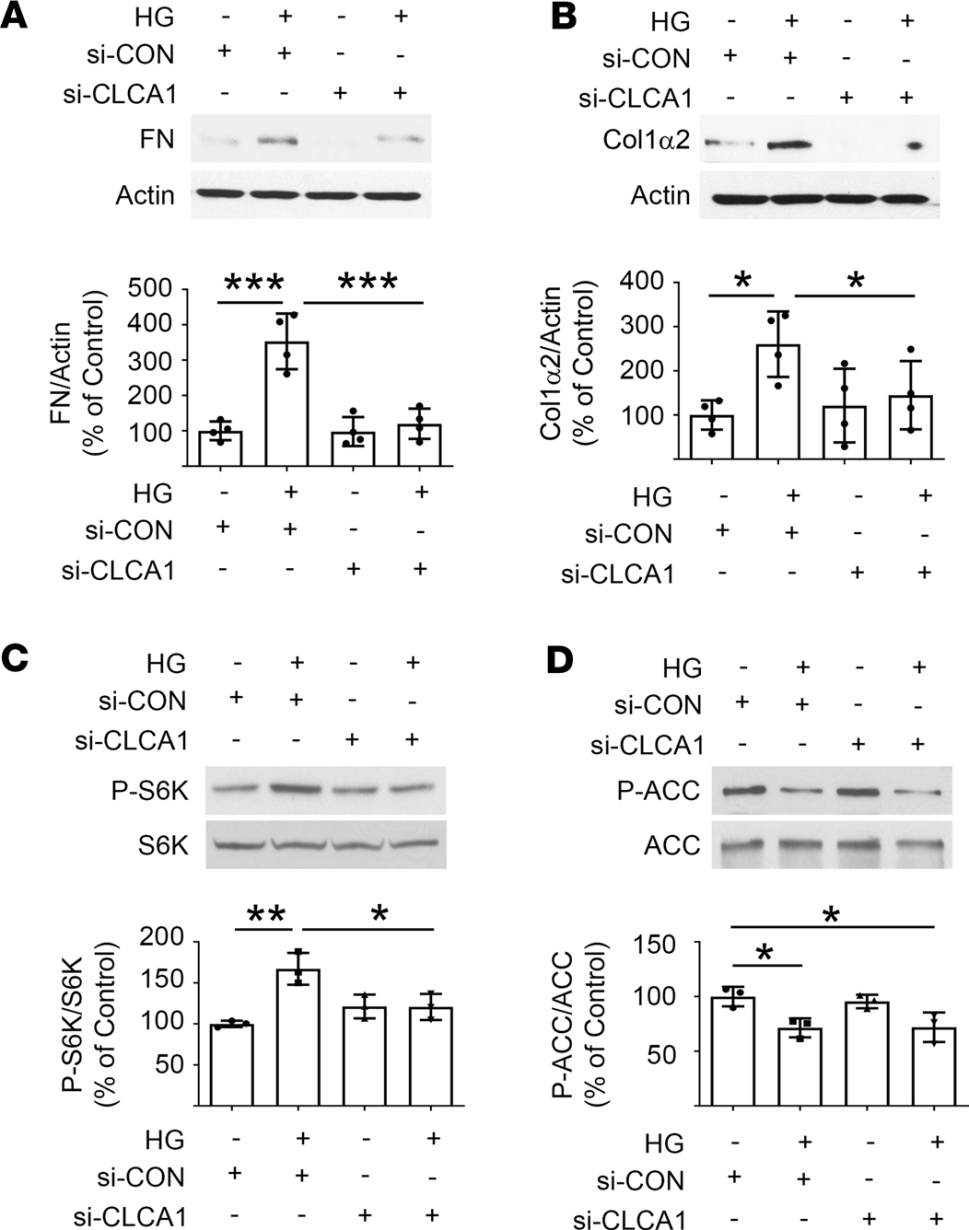
CLCA1 mediates high glucose–induced matrix protein synthesis in proximal tubular epithelial cells. (**A** and **B**) High glucose–induced increase in fibronectin and collagen 1α2 was inhibited by reduced expression of CLCA1. (**C** and **D**) siRNA-mediated reduced CLCA1 expression did not permit mTORC1 activation by high glucose; however, it did not affect high glucose inhibition of AMPK activity. ACC, acetyl-CoA carboxylase. Data from 3–4 experiments (mean ± SD) are shown in bars with scatterplots and were analyzed by ANOVA. *P < 0.05, **P < 0.01, ***P < 0.001.

Matrix expansion contributes importantly to fibrosis, leading to loss of kidney function in diabetes. We studied whether CLCA1 takes part in high glucose–induced matrix protein increase. siRNA-mediated reduction in CLCA1 expression (Supplemental Figure 1A; supplemental material available online with this article; https://doi.org/10.1172/jci.insight.174848DS1) significantly inhibited high glucose–induced increase in fibronectin (Figure 4A) and collagen 1α2 expression at 24 hours (Figure 4B). Kidney matrix protein increase in diabetes is dependent on inhibition of AMPK activity that is linked to mTORC1 activation (30, 35, 36). We examined these signaling reactions. Reduced CLCA1 expression by siRNA abolished high glucose–induced mTORC1 activation (Figure 4C). In contrast, high glucose–induced reduction in AMPK activity was unaffected (Figure 4D). These data reveal a mechanism in which CLCA1 is required for high glucose–induced mTORC1 activation and matrix protein increase in proximal tubular epithelial cells; however, CLCA1 does not appear to be involved in high glucose regulation of AMPK.

### Regulation of TMEM16A by high glucose.

**Figure 5:**
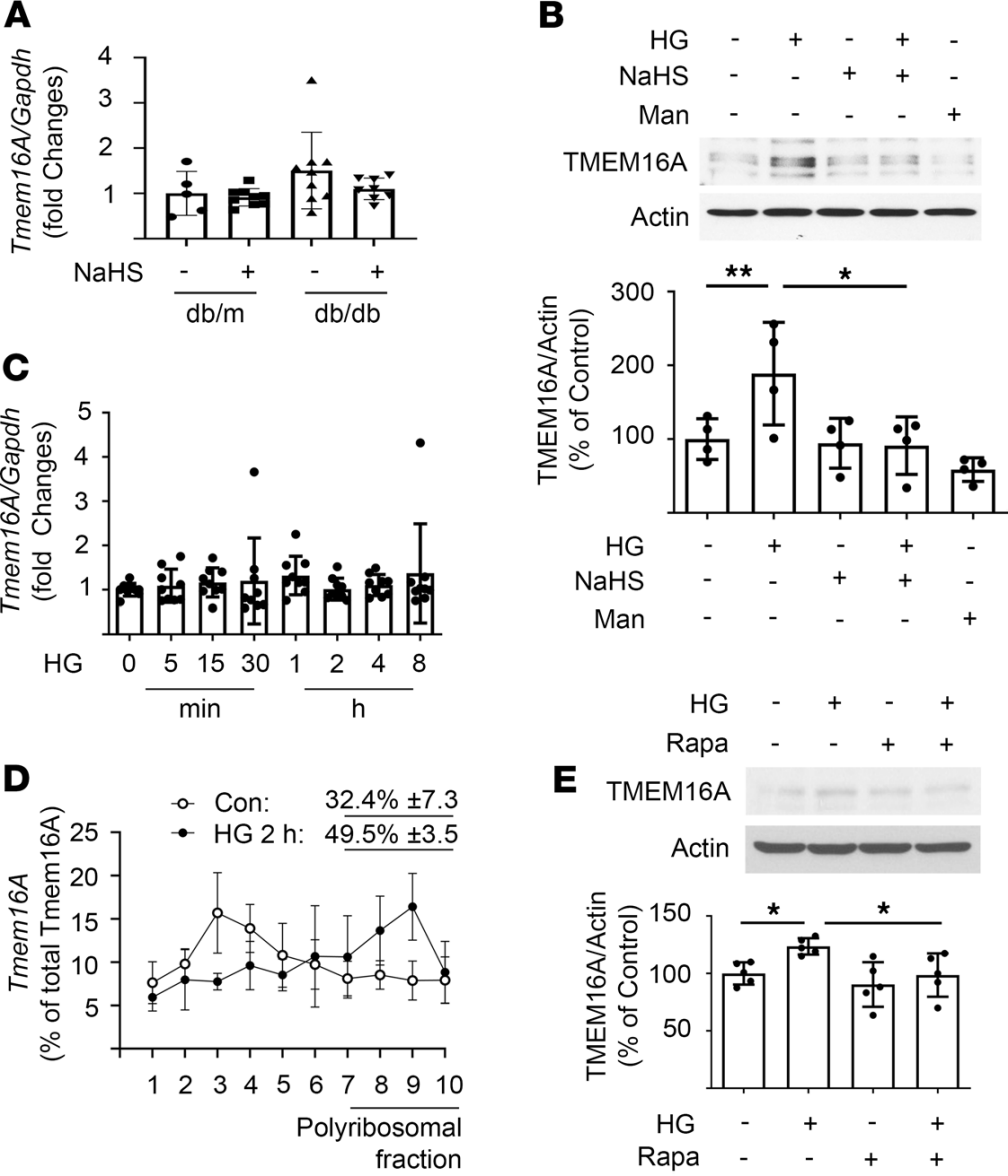
High glucose regulation of TMEM16A. (**A**) *Tmem16A* mRNA content was unchanged in the renal cortical lysates from diabetic mice as measured by qRT-PCR; it was unaffected by NaHS (*n* = 5–9 mice per group). (**B**) High glucose but not equimolar mannitol (Man) increased TMEM16A protein expression at 2 hours in proximal tubular epithelial cells that was abolished by NaHS. (**C**) qRT-PCR showed that high glucose did not affect *Tmem16A* expression. (**D**) Polyribosomal assay demonstrated that high glucose increased distribution of *Tmem16A* mRNA to the polyribosomal fractions (*P* < 0.05 by *t* test), which would facilitate increase in its translation. (**E**) Rapamycin, an inhibitor of mTOR, abrogated increase in TMEM16A expression induced by high glucose. (**A**–**C** and **E**) Data from 4–8 experiments (mean ± SD) are shown in bars with scatterplots and were analyzed by ANOVA. *P < 0.05, **P < 0.01.

Increase in renal cortical TMEM16A protein expression in diabetic mice (Figure 1A) was not associated with changes in its mRNA content (Figure 5A), suggesting nontranscriptional regulation. High glucose, but not equimolar mannitol, increased TMEM16A expression at 2 hours in MCT cells that was inhibited by NaHS (Figure 5B). *Tmem16A* mRNA content in cells did not change with high glucose (Figure 5C), aligning with data in diabetic mice (Figure 5A). We tested if mRNA translation could be the site of regulation by examining whether there was an increased association of *Tmem16A* mRNA with polyribosomes. Polyribosomal *Tmem16A* mRNA content was increased in high glucose–treated cells compared with controls (Figure 5D). Because mTORC1 governs mRNA translation (37), we studied the effect of rapamycin, its inhibitor. Rapamycin abolished high glucose–induced TMEM16A expression, indicating mediation by mTOR (Figure 5E). These data show that high glucose regulates CLCA1 and TMEM16A expression by distinct mechanisms in mice. In humans, increased tubular *TMEM16A* mRNA was associated with a trend toward increase in its protein, suggesting a transcriptional regulation (Figure 1, H and J). We further examined this possibility. High glucose increased TMEM16A protein expression in a time-dependent manner in human kidney proximal tubule (HK2) cells; however, this was not accompanied by increase in its mRNA (Supplemental Figure 2, A and B). It is possible that factors involved in regulation of *TMEM16A* transcription in the human kidney are lost in HK2 cells in culture. Together these data suggest that TMEM16A regulation is different in humans compared with mice.

### TMEM16A mediates high glucose induction of matrix protein expression.

**Figure 6:**
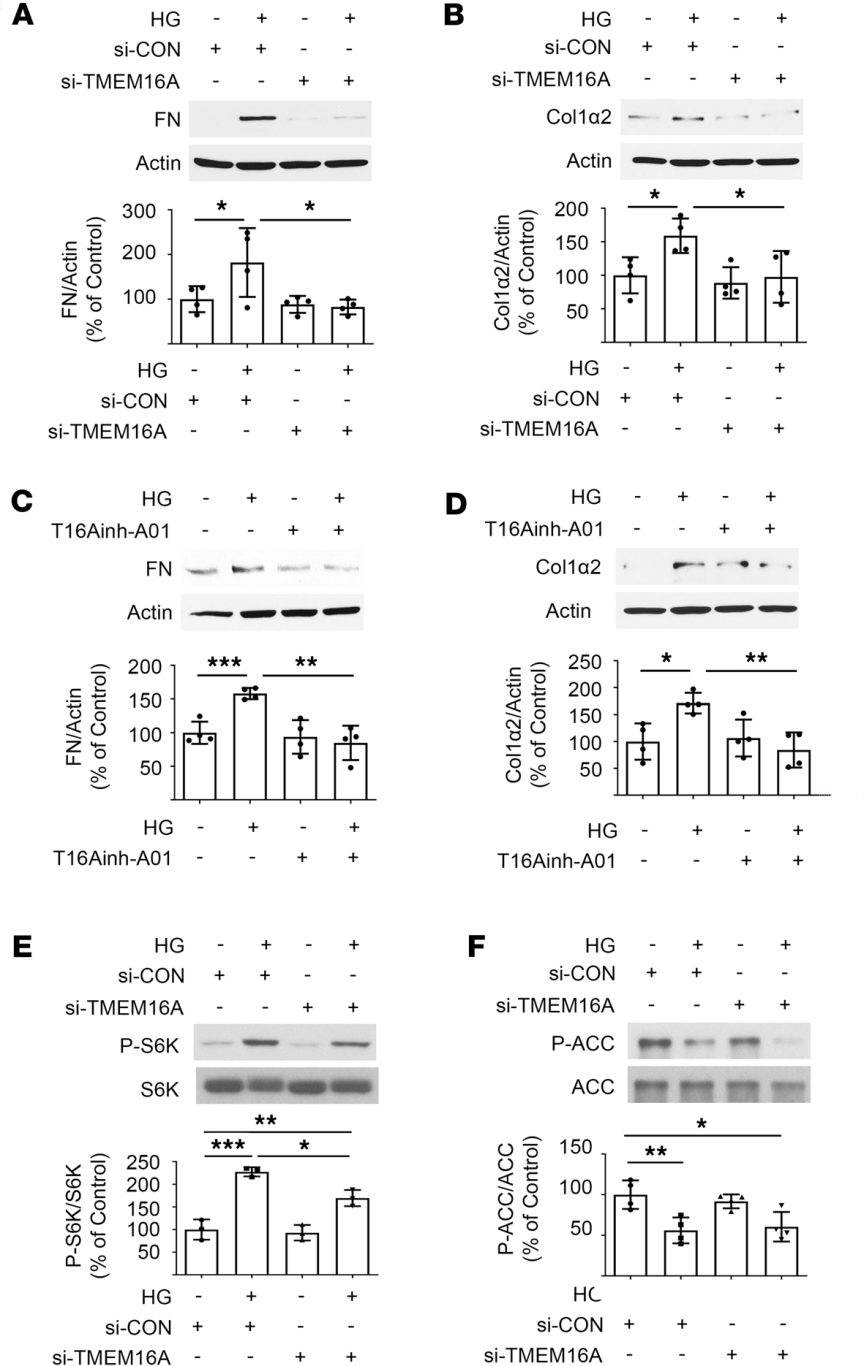
TMEM16A mediates high glucose–induced matrix proteins in proximal tubular epithelial cells. (**A** and **B**) siRNA against TMEM16A abolished high glucose–induced increased expression of fibronectin and collagen 1α2. (**C** and **D**) TMEM16A inhibitor, T16Ainh-A01, also suppressed high glucose–stimulated expression of fibronectin and collagen 1α2. (**E** and **F**) TMEM16A siRNA partly abolished mTORC1 activation by high glucose but not high glucose inhibition of AMPK activity. Data from 3–4 experiments (mean ± SD) are shown in bars with scatterplots and were analyzed by ANOVA. *P < 0.05, **P < 0.01, ***P < 0.001.

siRNA-mediated reduction in TMEM16A (Supplemental Figure 1B) abolished increased fibronectin and collagen 1α2 in high glucose–treated cells (Figure 6, A and B). Similar results were obtained upon employing T16Ainh-A01, a chemical inhibitor of TMEM16A (Figure 6, C and D). These data show that high glucose–induced increase in matrix proteins in proximal tubule cells requires TMEM16A. Reduction in TMEM16A expression by siRNA partly abrogated high glucose induction of mTORC1 activation (Figure 6E) but not the reduction in AMPK activity (Figure 6F). Together with observations with CLCA1 (Figure 4D), these data suggest that the CLCA1/TMEM16A axis is required for high glucose stimulation of mTORC1 but not for regulation of AMPK.

### High glucose stimulates Cl^–^ current through TMEM16A; it is controlled by H_2_S.

**Figure 7:**
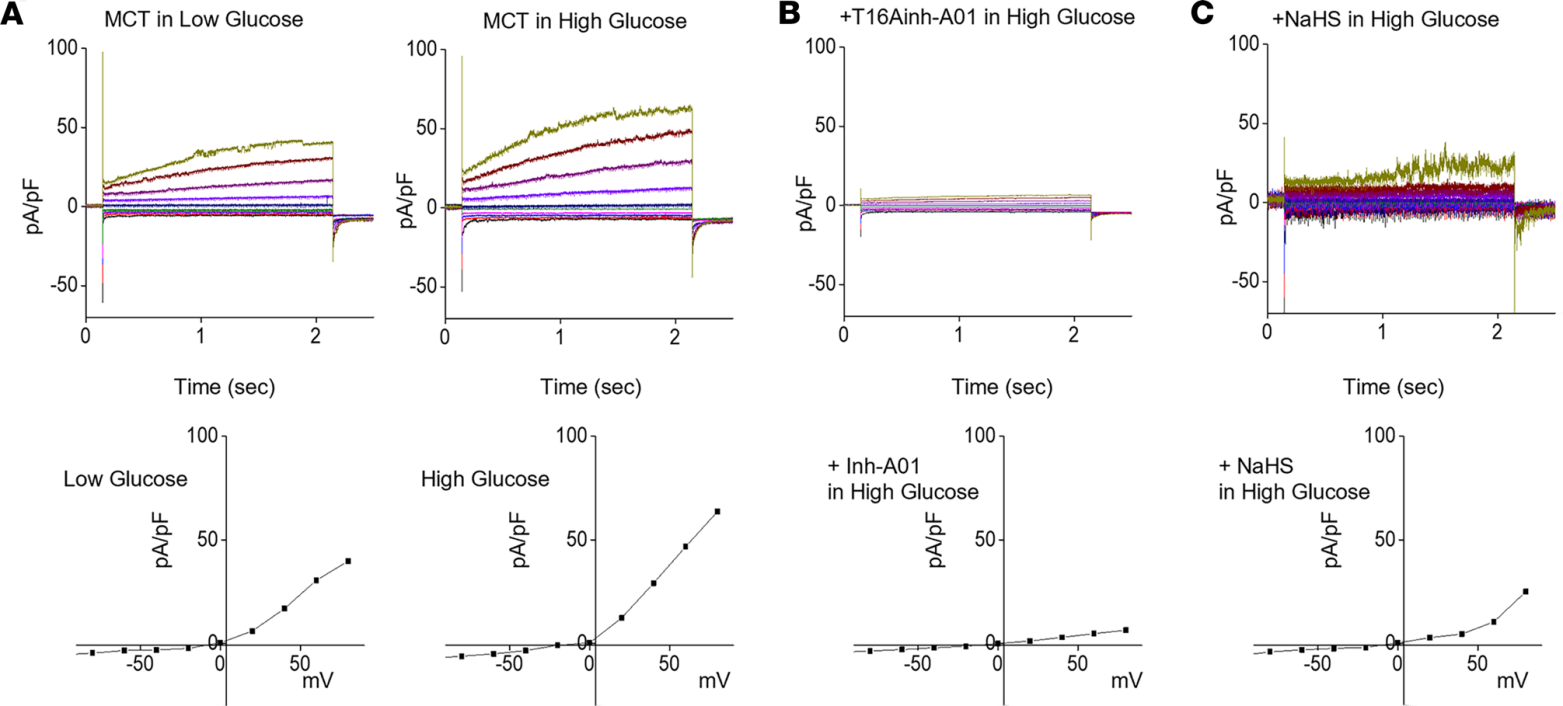
High glucose stimulates Cl^–^ current by TMEM16A that is inhibited by H_2_S. (**A**) Relative to normal glucose, high glucose increased Cl^–^ current as determined by patch clamp. (**B** and **C**) High glucose–induced Cl^–^ current was abrogated by T16inh-A01, an inhibitor of TMEM16A, and by NaHS.

Although CLCA1 and TMEM16A are required for high glucose stimulation of mTORC1 and matrix protein expression, we do not know if it involves their composite regulation of Cl^–^ current. Accordingly, we studied Cl^–^ current by performing whole-cell patch clamp. Compared with normal glucose, Cl^–^ current was increased by high glucose in MCT cells (Figure 7A). High glucose–induced Cl^–^ current was abolished by TMEM16Ainh-A01, verifying identification of the Cl^–^ channel as TMEM16A (Figure 7B). NaHS abolished high glucose–induced Cl^–^ current (Figure 7C), demonstrating H_2_S deficiency (Figure 1B) leads to high glucose stimulation of the CLCA1/TMEM16A/Cl^–^ current axis.

TMEM16A is a Ca^2+^-dependent Cl^–^ channel. High glucose increases intracellular Ca^2+^ by the store-operated Ca^2+^ entry (SOCE) mechanism in kidney cells (38). TMEM16A activation is dependent on ORAI-1, a component of SOCE (39). High glucose but not equimolar mannitol augmented intracellular Ca^2+^. Additionally, Cl^–^ current was inhibited by an SOCE inhibitor (Supplemental Figure 3, A and B) when compared with high glucose (Figure 7A). These data show that high glucose augmented Ca^2+^ transient by the SOCE mechanism and that the downstream Cl^–^ current is SOCE dependent. Because equimolar mannitol did not affect the Ca^2+^ transient, high glucose regulation of intracellular Ca^2+^ is not due to its osmotic effect.

## Discussion

**Figure 8:**
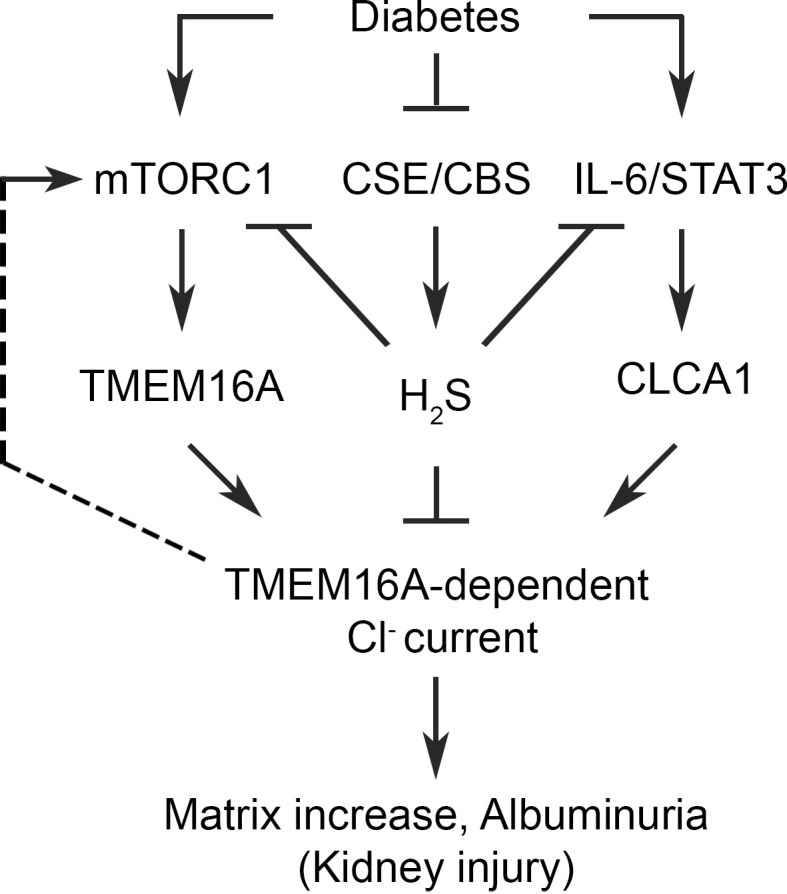
A schematic summarizes the role of the CLCA1/TMEM16A/Cl^–^ current in diabetes-associated kidney injury.

Our investigation shows the following: (a) Diabetes is associated with increase in the kidney expression of CLCA1 and TMEM16A, resulting in increased Cl^–^ current; (b) CLCA1/TMEM16A/Cl^–^ current system participates in stimulation of mTOR and increased matrix protein expression (Figure 8).

Mechanisms by which diabetes regulates renal CLCA1 and TMEM16A expression in mice are distinct. Our investigations demonstrated that augmented transcription mediated by IL-6/STAT3 accounts for high glucose–induced increase in CLCA1 in mice. Interestingly, inhibition of STAT3 ameliorates kidney injury in diabetic mice (40).

In contrast with CLCA1, a nontranscriptional mechanism seemed to underlie high glucose–induced increase in TMEM16A in mice involving greater efficiency of mRNA translation. Synthesis of select kidney proteins, such as laminin, vascular endothelial factor, and CSE, is independently regulated by translation (30, 41–45). We explored the mechanism underlying augmented *Tmem16A* mRNA translation. mTORC1 activates mRNA translation in diabetic kidney injury (30, 37, 46, 47). In type 2 diabetic mice, renal tissue laminin increase is regulated at the level of mRNA translation and could be mitigated by rapamycin, an mTOR inhibitor (30, 48). In the current study, rapamycin abolished high glucose–induced TMEM16A expression, suggesting its translation is controlled by mTORC1. In contrast, in human DKD, increase in tubular *TMEM16A* is associated with increase in its protein expression, suggesting a transcriptional mechanism that differs from its regulation in mice. These data suggest that control of expression of CLCA1 and TMEM16A genes varies between mice and humans. First, difference in genera could be involved. Studies comparing genomics of mice and humans have shown that there are important variations in transcription, DNase I hypersensitivity, transcription factor binding, chromatin modifications, and replication domains (49). These differences could alter how a candidate gene is regulated, i.e., transcription versus mRNA translation. If the regulatory elements for binding of transcription factors to mouse *Clca1* gene are favorable, it could lead to a transcriptional mechanism for its expression in mice but not in humans. This would explain *Clca1* mRNA increase in mice but not in humans. In a similar vein, it is possible that transcriptional mechanisms are favored in the tubules of kidneys in humans whereas the regulatory environment in mice may favor mRNA translation as a mechanism of increase in TMEM16A. Further studies are needed to evaluate these potential mechanisms. Additionally, even without modification of its protein expression, it is possible that the *activity* of CLCA1 could still be stimulated in humans, resulting in potentiation of Cl channel activity by TMEM16A.

Our in vitro studies with siRNA or selective inhibitor of TMEM16A revealed an interesting observation that the CLCA1/TMEM16A/Cl^–^ current system is required for high glucose–induced mTORC1 signaling and matrix protein increase. Relevant to diabetic kidney injury, high glucose activates the PI3K/Akt/mTORC1 axis in the kidney (35, 44, 50–52). Our in vitro findings suggest that high glucose stimulation of mTORC1 is at least partly mediated by the CLCA1/TMEM16A/Cl^–^ current system (Figure 8). Inhibition of TMEM16A resulted in mitigation of renal fibrosis in unilateral ureteral obstruction, showing its requirement for kidney injury (22).

The mechanism by which Cl^–^ secretion results in downstream matrix protein synthesis is unclear. Shift of fluid across cell membrane accompanying Cl^–^ secretion is likely to result in change in cell shape and induce mechanical stress, which may transmit cues downstream by mechanotransduction. In podocytes mechanotransduction induced by increased glomerular filtrate flow-associated shear stress leads to Akt activation (53). Akt directly activates mTORC1 and protein synthesis (37). Additionally, cell shape change may activate focal adhesion kinase (FAK). FAK activates the PI3K/Akt axis (54). Renal FAK activation occurs in DKD (55). Thus, FAK activation can stimulate the PI3K/Akt/mTORC1 axis. Since mTORC1 is a master regulator of protein synthesis, its stimulation leads to augmented synthesis of matrix proteins and kidney fibrosis in diabetic kidney injury. Additionally, Cl^–^ secretion disorders are associated with inflammation, increased oxidative stress, and mitochondrial dysfunction, many of which stimulate mTORC1 and impair autophagy (56). Our current data show that augmented Cl^–^ secretion by TMEM16A and activated mTORC1 occur in association with increased expression of IL-6, a pro-inflammatory cytokine, in diabetic kidney injury (Figure 3). IL-6 regulates CLCA1 expression via STAT3 (Figure 3). Thus, in diabetes, signals from increased Cl^–^ secretion by the CLCA1/TMEM16A system lead to activation of mTORC1, resulting in increased synthesis of proteins, including matrix proteins. These potential pathways need further experimental evaluation.

Additional mechanisms may trigger PI3K/Akt signaling. Both the expression of TMEM16A and its function are amplified in a variety of malignancies, including those of breast (57–61), ovary (62), head and neck (63, 64), colon (65), and stomach (66). In select tumors, TMEM16A stimulation is linked to EGF receptor, which, in turn, stimulates signaling by PI3K/Akt (57).

Our findings in proximal tubule epithelial cells verify that high glucose increases intracellular Ca^2+^ by SOCE as reported in kidney mesangial cells (38). TMEM16A activation is dependent on ORAI-1, a component of SOCE (39). Interestingly, CLCA1 has been reported to increase SOCE (67). Thus, there could be 2 mechanisms by which CLCA1 stimulates TMEM16A function, i.e., by stabilizing it on the cell membrane, and, by increasing SOCE.

Interrogation of published gene expression data in kidney biopsies of humans showed that chronic kidney diseases of diverse etiology are associated with increased expression of *CLCA1* and *TMEM16A* (Supplemental Figure 4) (68). Tubulointerstitial fibrosis in people with IgA glomerulonephritis positively correlated with tubular TMEM16A expression (22). Thus, our findings are of relevance to both diabetes and other etiologies of chronic kidney disease in humans.

Emerging evidence supports an important role for H_2_S in the regulation of kidney injury in diabetes. Our data show that H_2_S deficiency in the kidney in diabetes is due to reduced enzymatic synthesis (27, 69, 70). Diabetes-induced H_2_S deficiency results in oxidative stress, matrix protein increase, and albuminuria, all of which can be mitigated by administration of H_2_S-releasing agents (26, 28, 70, 71). In the current study, H_2_S inhibited high glucose–induced increase in CLCA1 and TMEM16A in mouse MCT cells by inhibiting transcription and mRNA translation, respectively. This indicates H_2_S acts on early events in protein synthesis. Additionally, as we have reported previously, H_2_S inhibits mTORC1-mediated protein synthesis in high glucose–treated kidney cells (27). Inhibition of increased CLCA1, TMEM16A and Cl^–^ current, signaling events, matrix protein increase, and albuminuria by NaHS indicates that H_2_S deficiency is a crucial proximal injurious event in DKD. Additionally, lack of H_2_S has been linked to fibrosis in the unilateral ureteral obstruction model, which was ameliorated by NaHS (72). A recent report of requirement of TMEM16A for renal fibrosis in this model (22) suggests that H_2_S deficiency may be upstream of TMEM16A activation in obstructive nephropathy similar to our data in DKD. A limitation of our study is that the key initial mediators of glucotoxicity leading to regulation of H_2_S, CLCA1, TMEM16A, and mTOR were not interrogated.

In summary, the CLCA1/TMEM16A/Cl^–^ current axis is an important contributing mechanism in diabetic kidney injury that is triggered by deficiency in constitutive synthesis of H_2_S. Components of this axis, H_2_S, CLCA1, and TMEM16A, are attractive drug targets deserving further exploration in DKD.

## Methods

### Sex as a biological variable.

We excluded females in the current study because we have recently shown that female mice resist diabetes-induced kidney injury (73); it could be relevant to diabetic kidney injury in female mice.

### Cell culture.

MCT cells (provided by Eric Neilson, Northwestern University, Chicago, Illinois, USA) were grown in Dulbecco’s modified Eagle’s medium containing 7% fetal bovine serum, 5 mM glucose, 100 units/mL penicillin, 100 μg/mL streptomycin, and 2 mM glutamine. Cells were incubated with high glucose (30 mM) or 5 mM glucose + 25 mM mannitol after 24-hour serum starvation (74).

### Polyribosome assay.

Assay was performed as previously described with some modification (75, 76). Briefly, postnuclear supernatants were separated on a 10%–50% sucrose gradient by centrifugation at approximately 200,000*g* and divided into 10 fractions; fractions 7–10 were considered to contain polyribosomes. Total RNA was isolated by the TRIzol method (Invitrogen, Thermo Fisher Scientific) and used for cDNA synthesis. The cDNA was used for qRT-PCR with *Tmem16A* primers (catalog, 330001; gene ID, PPM26917B-200; QIAGEN).

### Transfection with siRNA.

Cells were transfected with siRNA as described (27). Briefly, control siRNA-A (catalog, sc-37007, Santa Cruz Biotechnology) or pools of siRNA for *Clca1* (catalog, sc-142370, Santa Cruz Biotechnology) or *Tmem16A* (catalog, sc-76687, Santa Cruz Biotechnology) were diluted into siRNA transfection media (catalog, sc-36868, Santa Cruz Biotechnology) with Lipofectamine RNAiMAX transfection reagent (catalog, 13778150, Thermo Fisher Scientific). After transfection for 24 hours, cells were quiesced in serum-free media for 24 hours before performing experiments.

### Animals.

We employed 12-week-old male db/db mice (catalog, 000642; Jackson Laboratory) and their control db/m mice (catalog, 000642; Jackson Laboratory). Diabetic and control mice were randomized to receive NaHS- (30 μmol/L) containing drinking water or water alone for 3 weeks (*n* = 9 in each group).

### Urinary albumin-to-creatinine ratio and blood glucose measurement.

Analytical kits were used to measure albumin (catalog E101 and catalog E90-134, Bethyl Laboratories) and creatinine (catalog ADI-907-030A, Enzo Life Sciences) (77). Blood glucose concentration was measured by a glucometer (Ascensia Diabetes Care US) (77).

### Immunoblotting.

Equal amounts of protein lysates or renal cortical homogenates were analyzed by immunoblotting (75, 78). We employed antibodies against the following: CLCA1 (catalog, ab180851, Abcam), TMEM16A (catalog, ab72984, Abcam), fibronectin (catalog, ab2413, Abcam), collagen 1α2 (catalog, 14695-1-AP, Proteintech Group), laminin-γ1 (catalog, sc-5584, Santa Cruz Biotechnology), phospho-Thr389-p70 S6 kinase (catalog, 9205, Cell Signaling Technology), p70 S6 kinase (catalog, 9202, Cell Signaling Technology), IL-6 (catalog, ab7737, Abcam), phospho-Tyr705-STAT3 (catalog, ab76315, Abcam), STAT3 (catalog, ab68153, Abcam), and actin (catalog, A2066, MilliporeSigma).

### Cl^–^ current measurement.

MCT cells were incubated with high glucose for 2 hours with T16Ainh-A01 (preincubation for 30 minutes) or NaHS (incubation at the same time with high glucose). Patch clamp experiments were performed as previously described (5).

### H_2_S measurement.

H_2_S content was measured by the monobromobimane method (79). Kidney cortical lysates were incubated with excess monobromobimane (catalog, B4380, MilliporeSigma) in 100 mM Tris-HCL buffer (pH 9.5, 0.1 mM diethylenetriaminepentaacetic acid: catalog, D6518, MilliporeSigma) for 30 minutes in 1% oxygen and at room temperature in a hypoxic chamber; the fluorescent product sulfide-dibimane was analyzed by reverse-phase HPLC employing an Agilent Eclipse XDB-C18 column (part number: 993967-902) and 0.1% trifluoroacetic acid (catalog, 28903, Thermo Fisher Scientific) in acetonitrile (catalog, 34851, MilliporeSigma) as the eluent. H_2_S generation in the kidney was measured as previously described (75).

### Gene expression profiling.

Biopsy samples for this study were from American Indians with type 2 diabetes who participated in a longitudinal study of diabetes and its complications (ClinicalTrials.gov number, NCT00340678). Annual measurements of glomerular filtration rate by the urinary clearance of iothalamate were recorded along with other lab measurements and morphometric assessments at the time of biopsy.

Kidney biopsy tissue procurement and gene expression profiling were performed on GeneChip Array Human Genome series U133A and Plus 2.0 (Affymetrix) as described previously (80–82). Affymetrix image files were obtained, processed, normalized, and batch corrected as described previously. In brief, the raw data were processed using Affy package and annotated with a custom CDF file in R statistical platform. After quantile normalization and batch correction using Comba, the intensities were log_2_-transformed and used in all downstream analysis.

### Statistics.

Data were expressed as mean ± SD; analyses between 2 groups were performed by 2-tailed *t* test using GraphPad Prism 8. Data were considered statistically significant at *P* < 0.05. Statistical comparisons between multiple groups were made by ANOVA 1-way analysis and post hoc analysis using Tukey’s multiple comparisons test employing GraphPad Prism 8; *P* < 0.05 was considered significant.

### Study approval.

Human study was approved by the Institutional Review Board of the National Institute of Diabetes and Digestive and Kidney Diseases of the NIH. Each participant signed an informed consent document. Additional experiments with human kidney tissue were approved by the University of Texas Health, San Antonio: IRB#: 20170635HU. Experiments employing mice were approved by the Institutional Animal Care and Use Committee of the University of Texas Health, San Antonio, and the South Texas Veterans Health Care System.

### Data availability.

There is no restriction on data availability. All data described in this report including supplemental materials are available in the report and the Supporting Data Values.

## Author contributions

BSK and HJL supervised the project in its conception, design, and data interpretation and wrote the manuscript. HJL and FD conducted most of the experiments. YS, SV, and BBS designed and conducted patch clamp experiments. RGN, MK, WJ, and VN helped in collecting and interpreting data on kidney samples from American Indians. CGK measured H_2_S content in kidney tissues. GGC, BBS, GZ, and KS participated in data interpretation and writing of the manuscript. All authors provided input and approved the manuscript.

## Supplementary Material

Supplemental data

Unedited blot and gel images

Supporting data values

## Figures and Tables

**Table 1:**
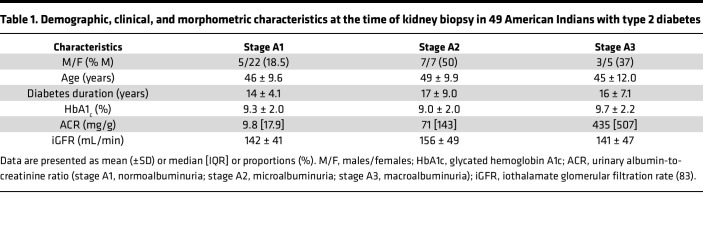
Demographic, clinical, and morphometric characteristics at the time of kidney biopsy in 49 American Indians with type 2 diabetes

## Version 1 01/09/2025

Electronic publication
