# Supplementary material for: The CLCA1/TMEM16A/Cl– current axis associates with H2S deficiency in diabetic kidney injury
Source: JCI Insight. 2025 Jan 9;10(1):e174848. doi: 10.1172/jci.insight.174848 (PMC11721299; doi:10.1172/jci.insight.174848)
Supplement: Supplemental data [file jciinsight-10-174848-s010.pdf]

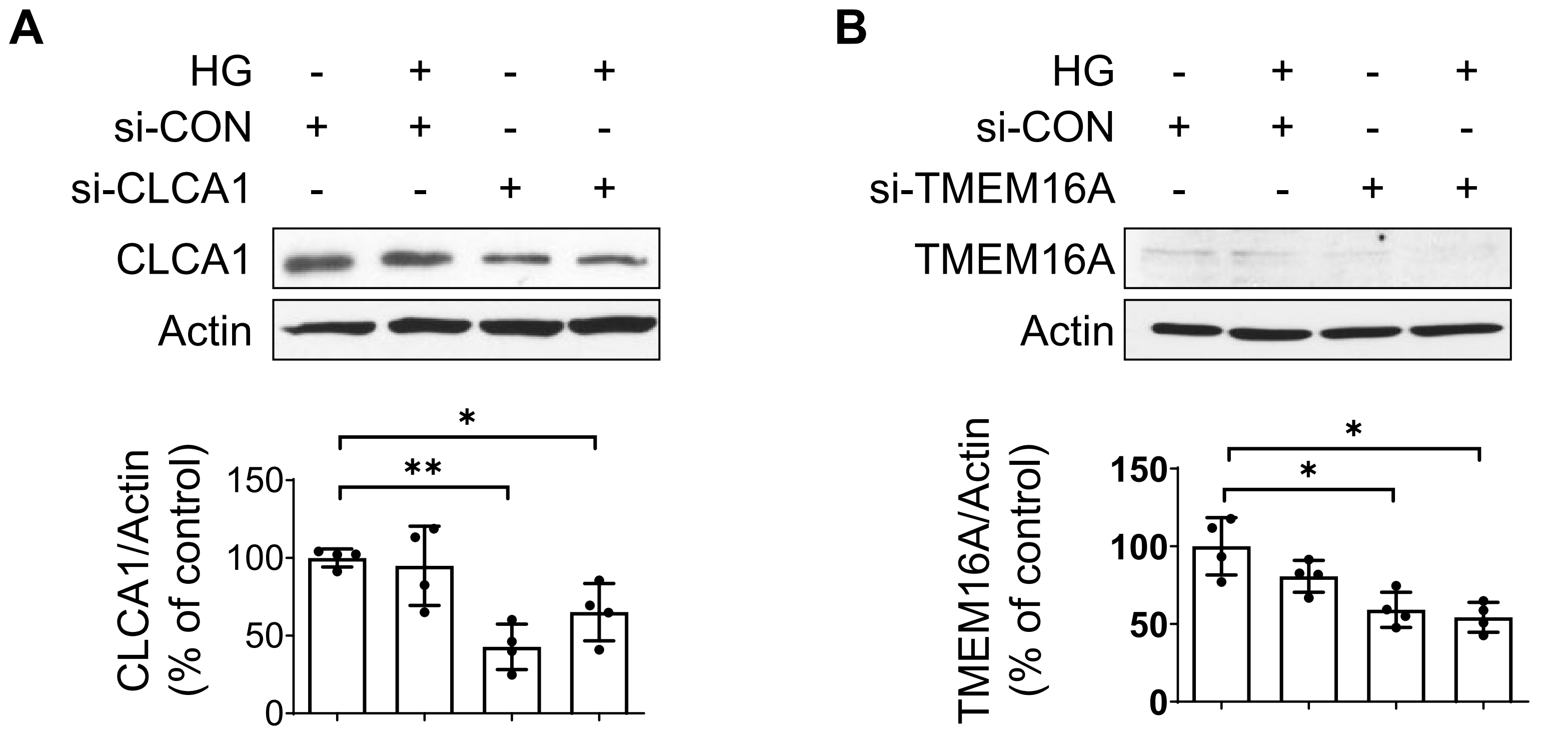

Suppl Fig. S1. A, B. Immunoblotting showed that siRNA against *Clca1* and *Tmem16a* reduced the corresponding protein expression in proximal tubule cells incubated with normal or high glucose. Representative blots from 4 experiments are shown,*$p<0.05$, **$p<0.01$ against normal glucose (lane 1).

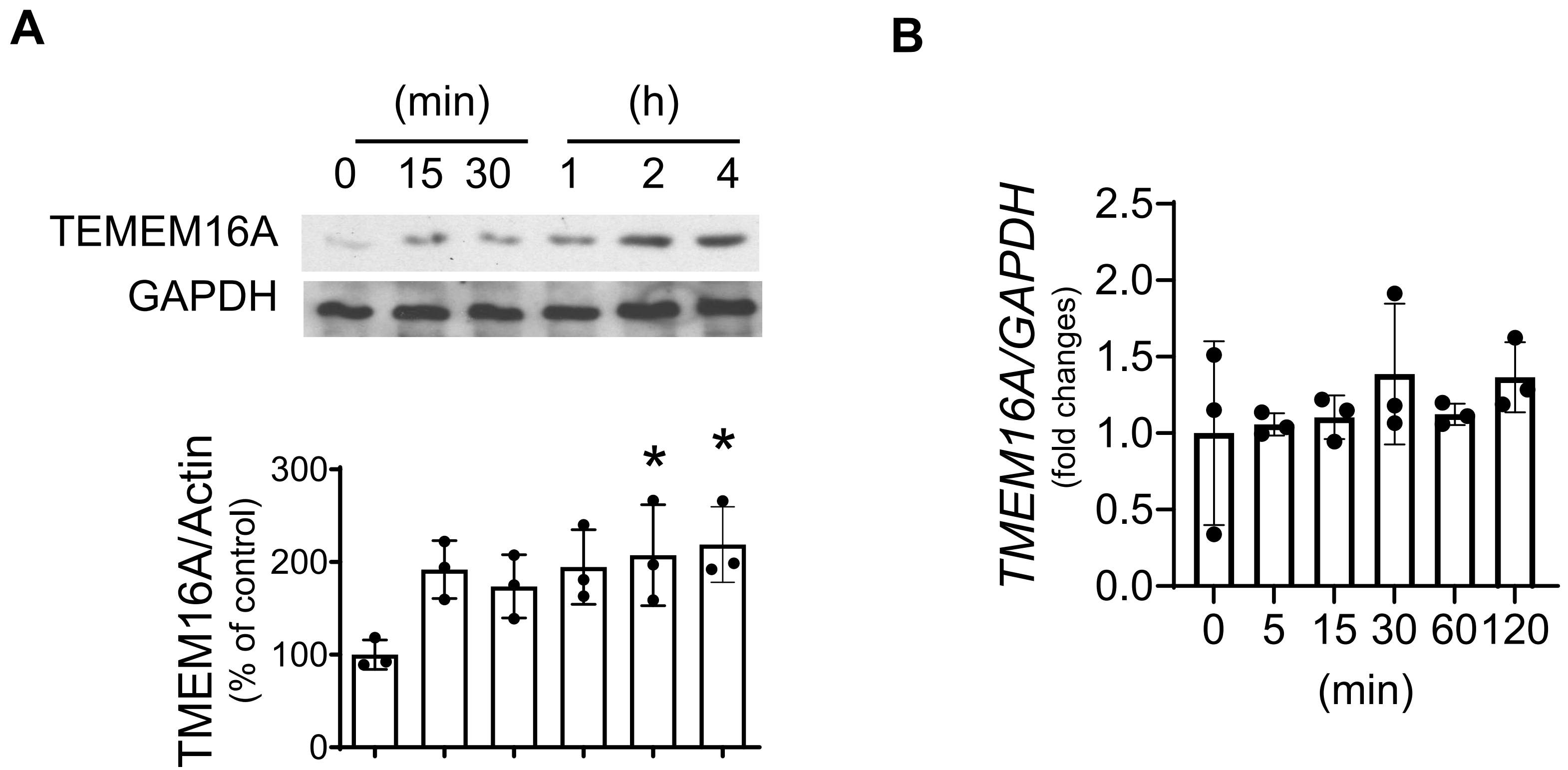

Suppl Fig. S2. HK2 (purchased from ATCC, CRL-2190) cells were maintained in DMEM/F12 media containing 7% fetal bovine serum, 5 mM glucose, 100 units/ml penicillin, 100 µg/ml streptomycin, and 2 mM glutamine. Cells were incubated with 30 mM glucose for the indicated time points. **A.** Immunoblotting showed that high glucose increased TMEM16A expression in HK2 cells, a human proximal tubule cell line. Representative blots from 4 experiments are shown, *p<0.05, **p<0.01 against normal glucose (lane 1). **B.** RT PCR showed that high glucose did not affect mRNA expression of TMEM16A in HK2 cells (3 experiments).

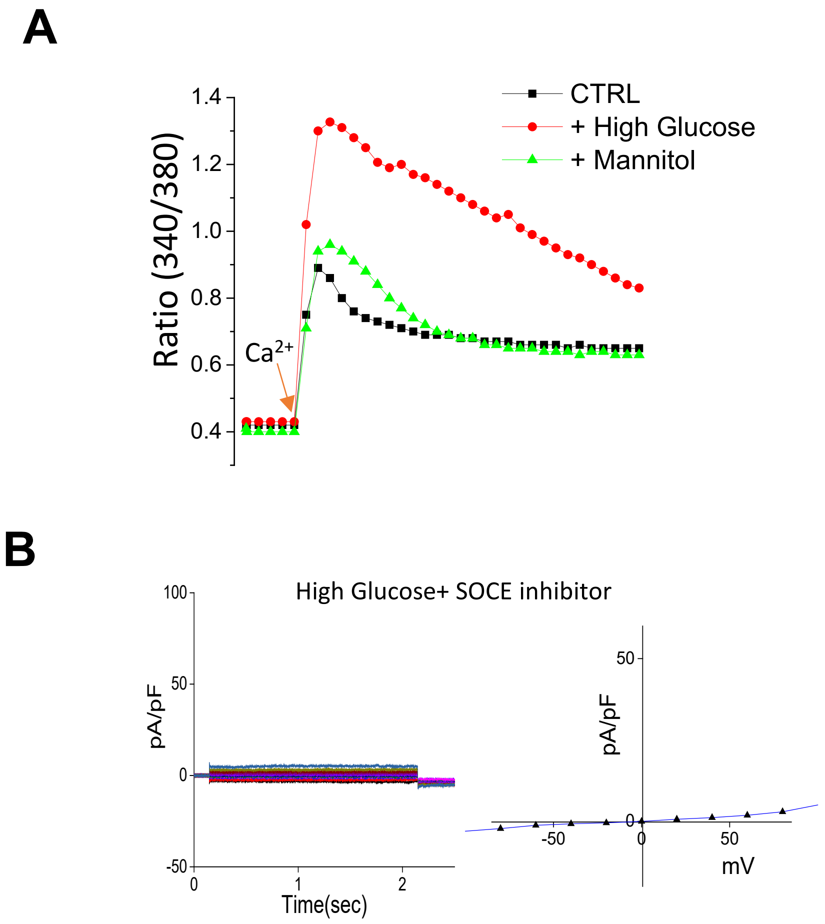

Suppl. Fig. S3. Effect of Store Operated Calcium Entry (SOCE) inhibitor on Cl^- current in MCT1 cells. For Ca^{++} measurements, the MCT cells were incubated with 2 μM Fura-2AM (Molecular Probes, USA) for 45 min after HG treatment. The images were obtained as previously described (Singh *et al*., *J Biol Chem* 2000. 275:36483–36486: Sun *et al*., 2017. *J Neurosci.* **37***,* 3364–3377). **A.** High glucose but not equimolar mannitol augmented intracellular Ca^{++}. **B.** SOCE inhibitor (10 μM, preincubation for 30 minutes) abolished Cl^- current induced by high glucose (compared with Fig. 7A).

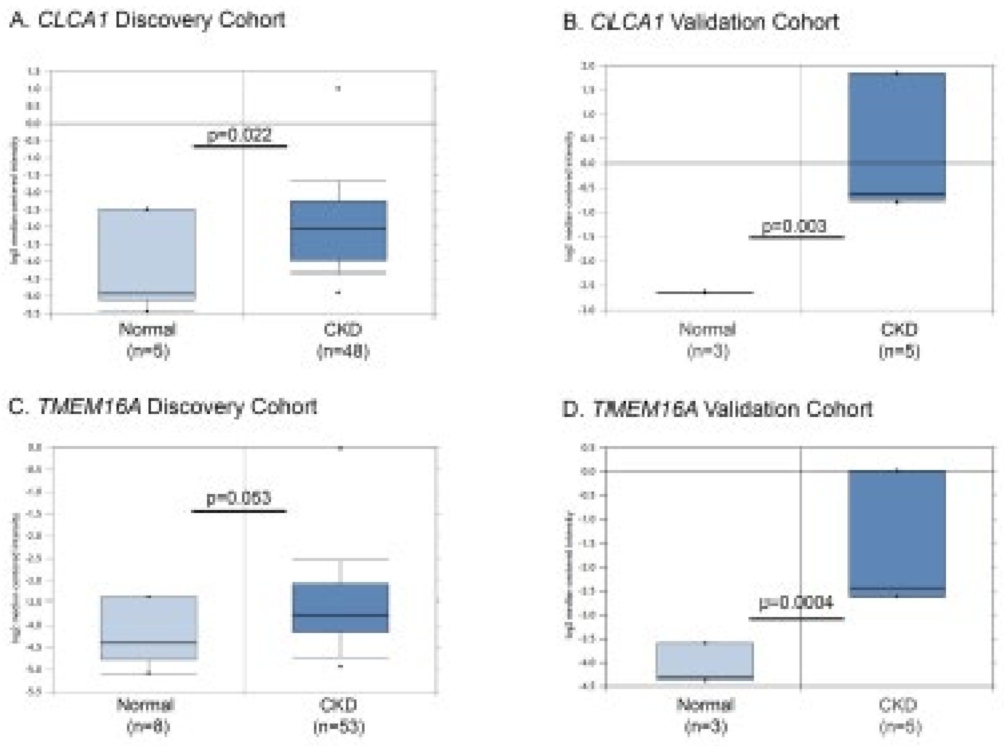

Suppl. Fig. S4. Gene expression of *CLCA1* and *TMEM16A* is increased in chronic kidney disease in human subjects. The publicly accessible Nephroseq dataset (nephroseq.org), The Regents of the University of Michigan, Ann Arbor, MI) was used for the expression analysis of CLCA1 and TMEM16A (Ano-1) in kidney biopsy samples. Specifically, we extracted the kidney gene expression comparison analyses between healthy controls and patients with CKD of diverse etiology from the Nakagawa study (Plos One. 2015, 10(8):e0136994) to test if there was a difference in the log2 median-centered intensity expression of *CLCA1* and *TMEM16A*.
