# Supplementary material for: The CLCA1/TMEM16A/Cl– current axis associates with H2S deficiency in diabetic kidney injury
Source: JCI Insight. 2025 Jan 9;10(1):e174848. doi: 10.1172/jci.insight.174848 (PMC11721299; doi:10.1172/jci.insight.174848)

Fig1A

Fig1A

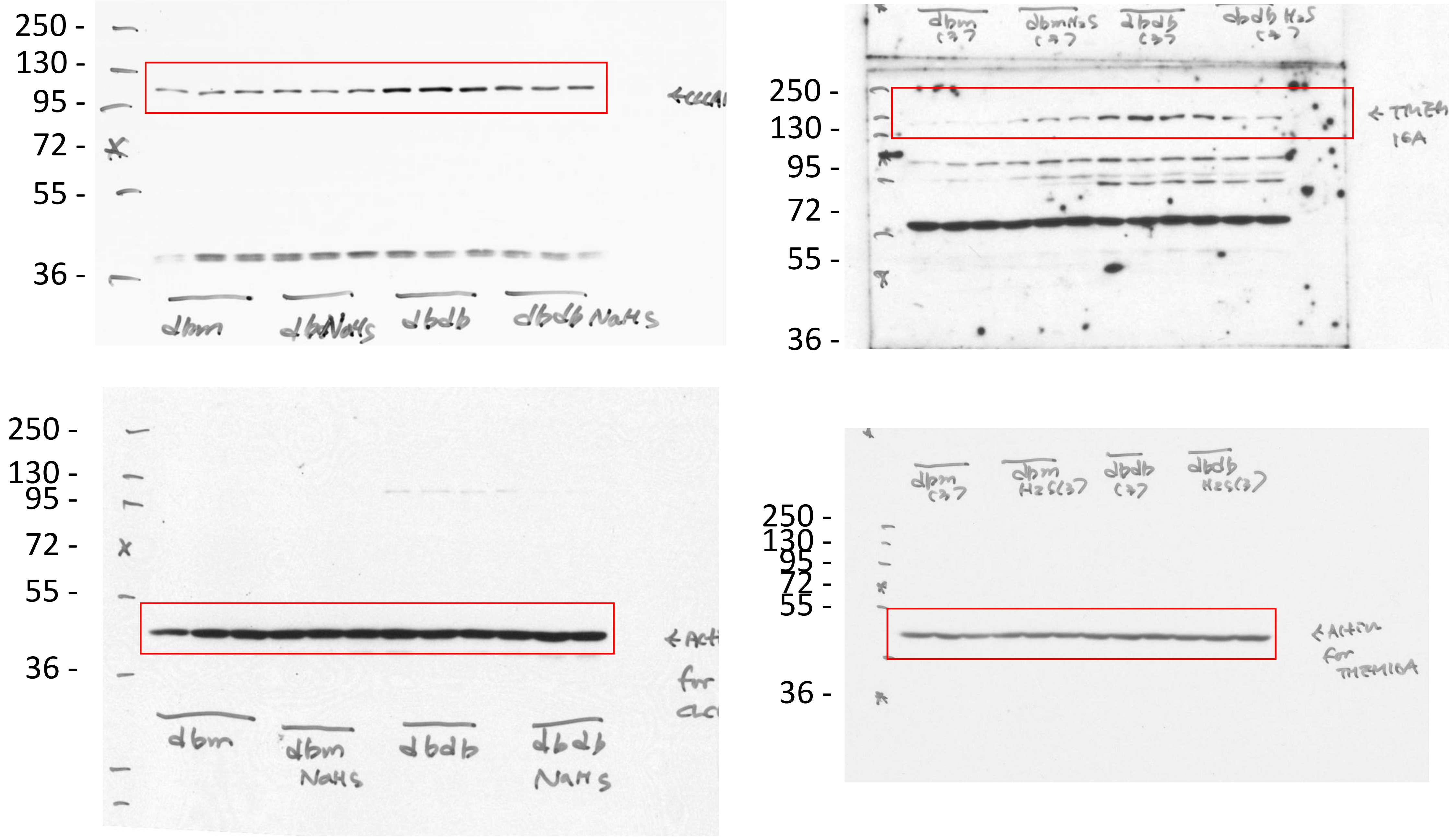

Fig1E

Fig1E

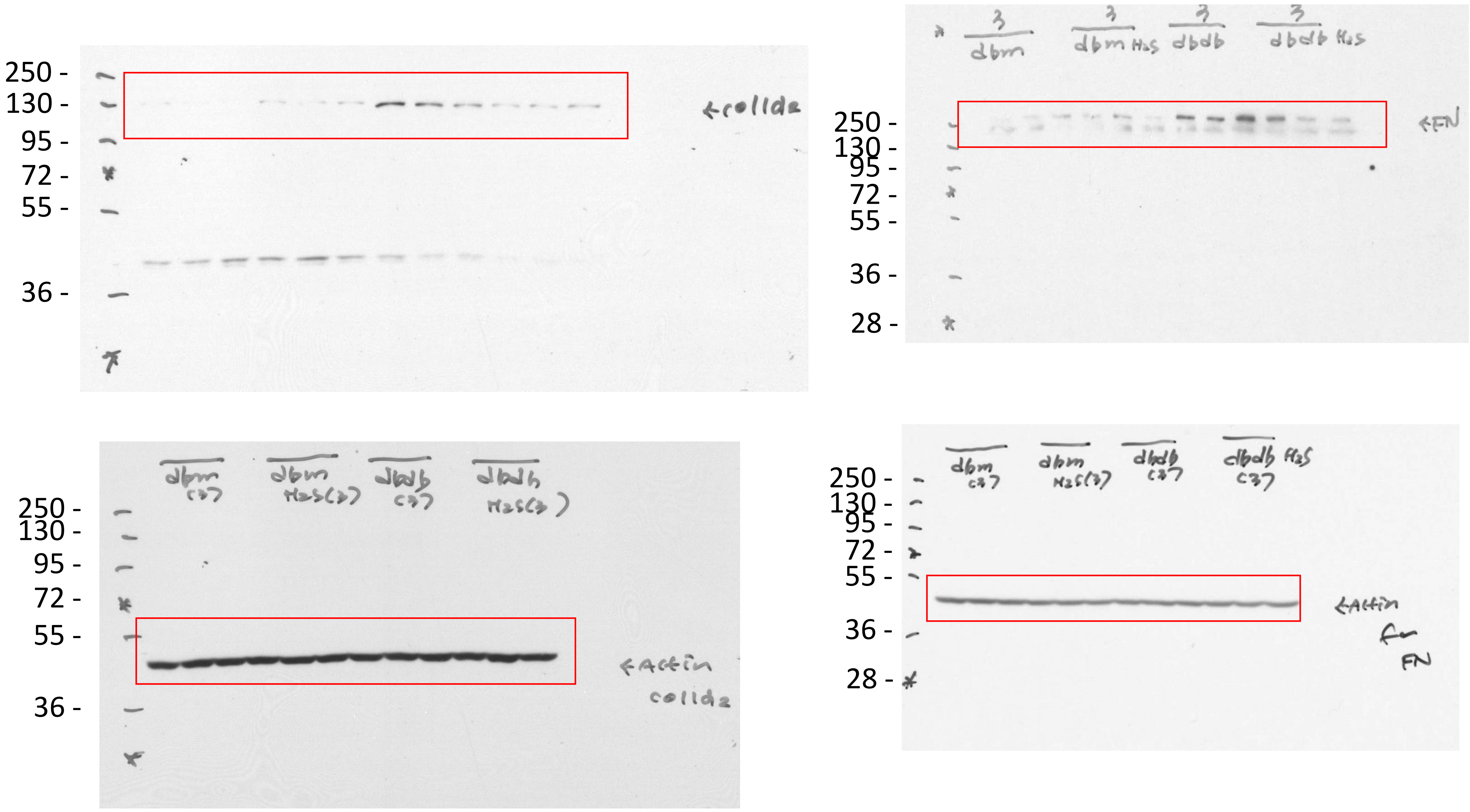

Fig1F

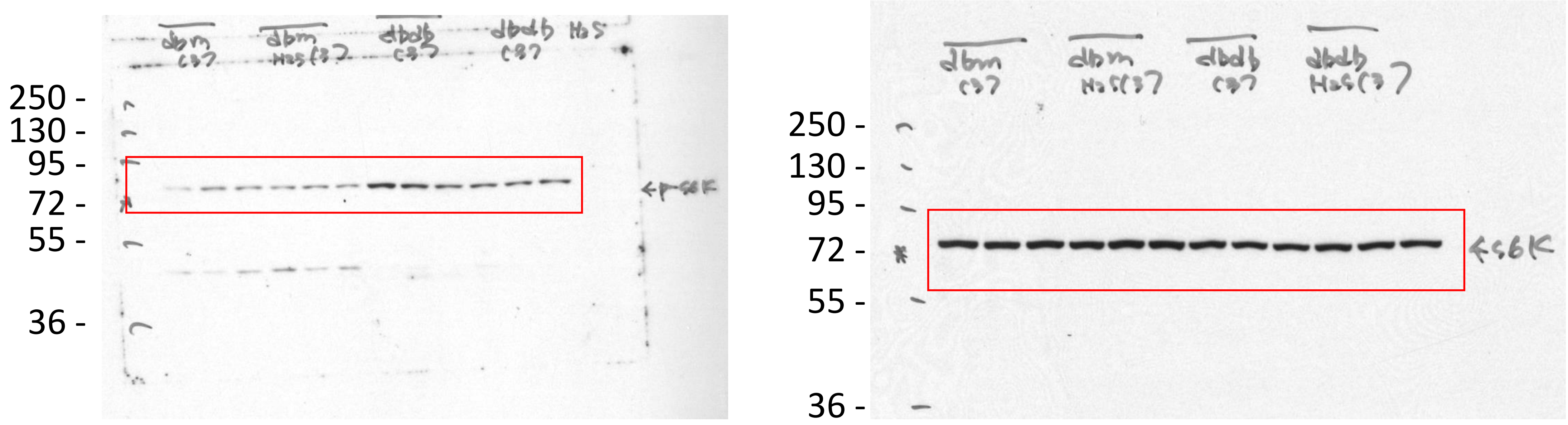

Fig1J

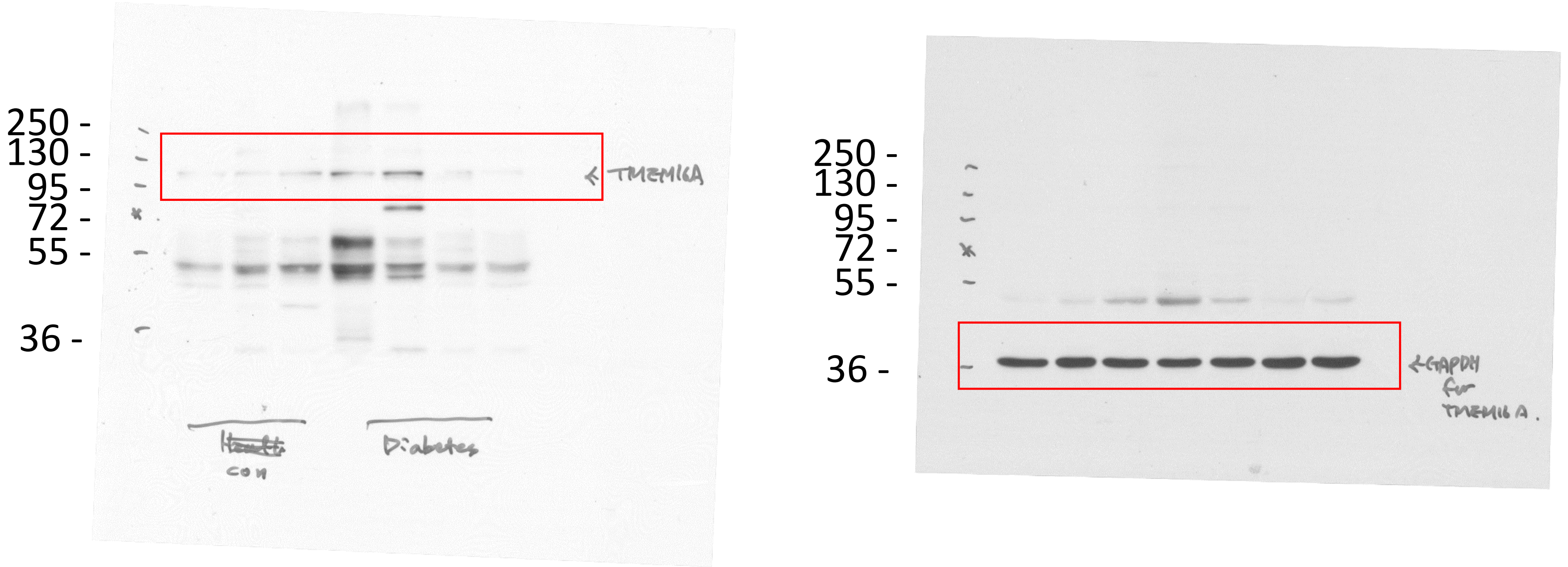

Fig2B

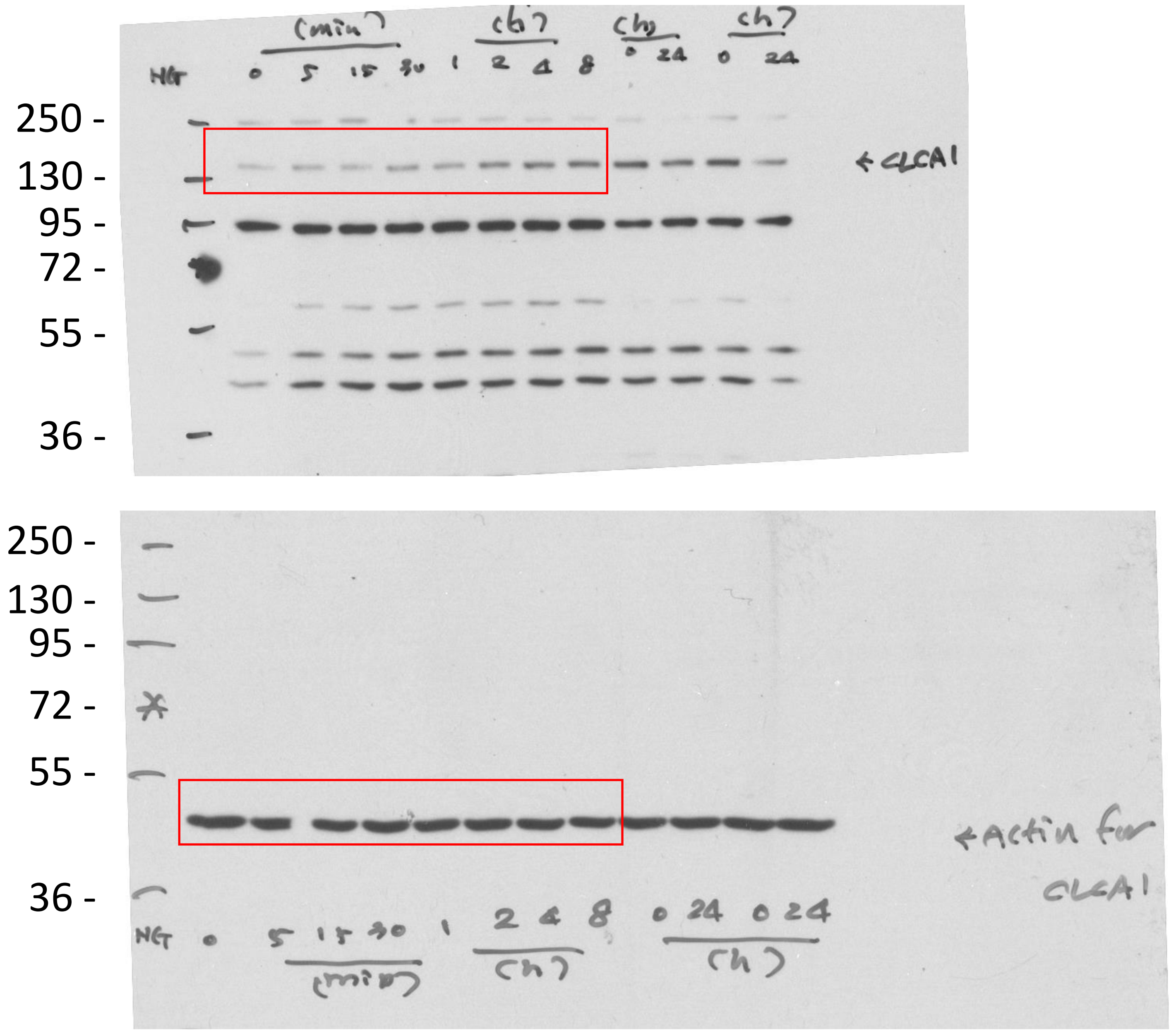
250 -
130 -
95 -
72 -
55 -
36 -
250 -
130 -
95 -
72 -
55 -
36 -

Fig2D

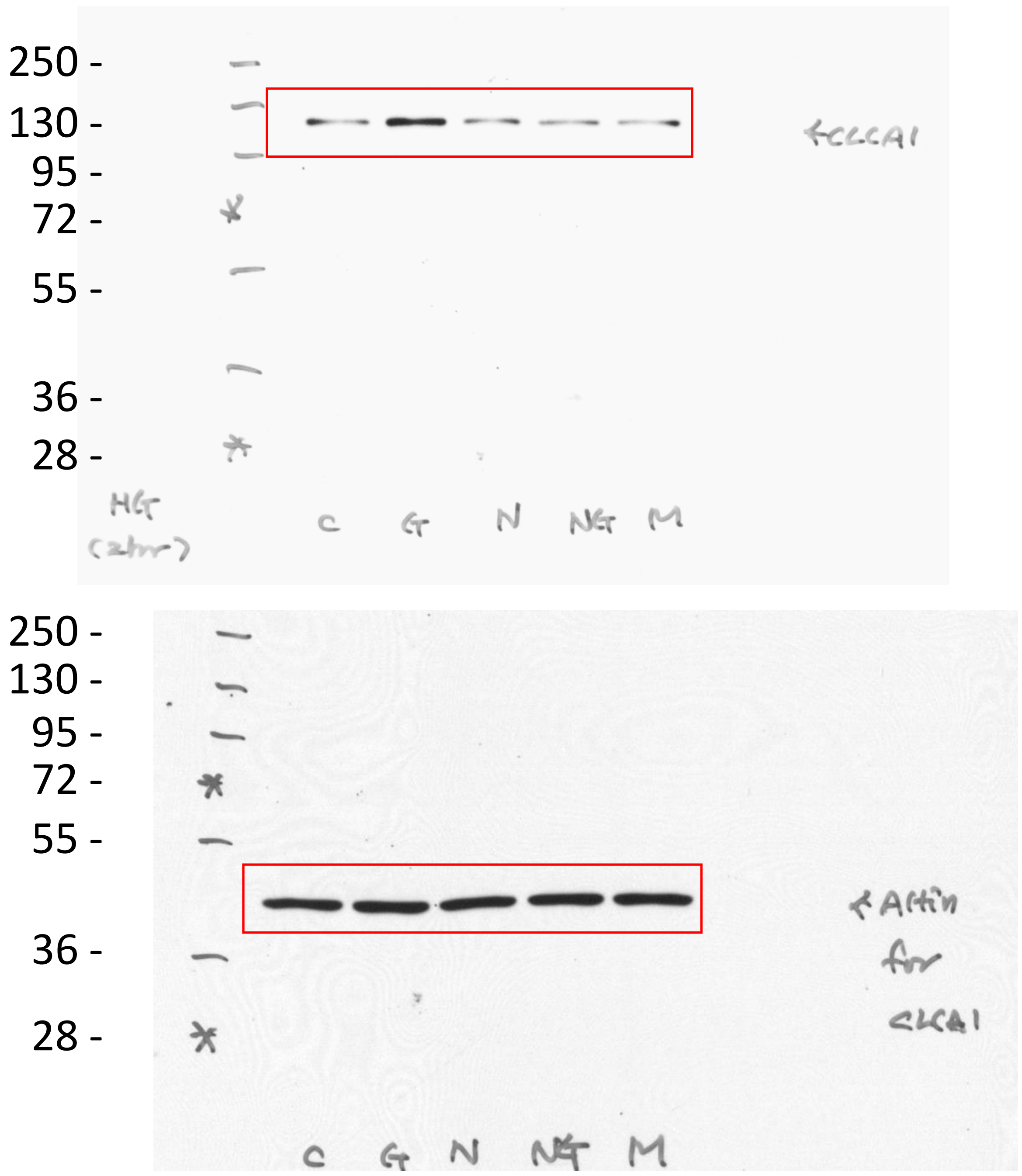
250 -
130 -
95 -
72 -
55 -
36 -
28 -
250 -
130 -
95 -
72 -
55 -
36 -
28 -

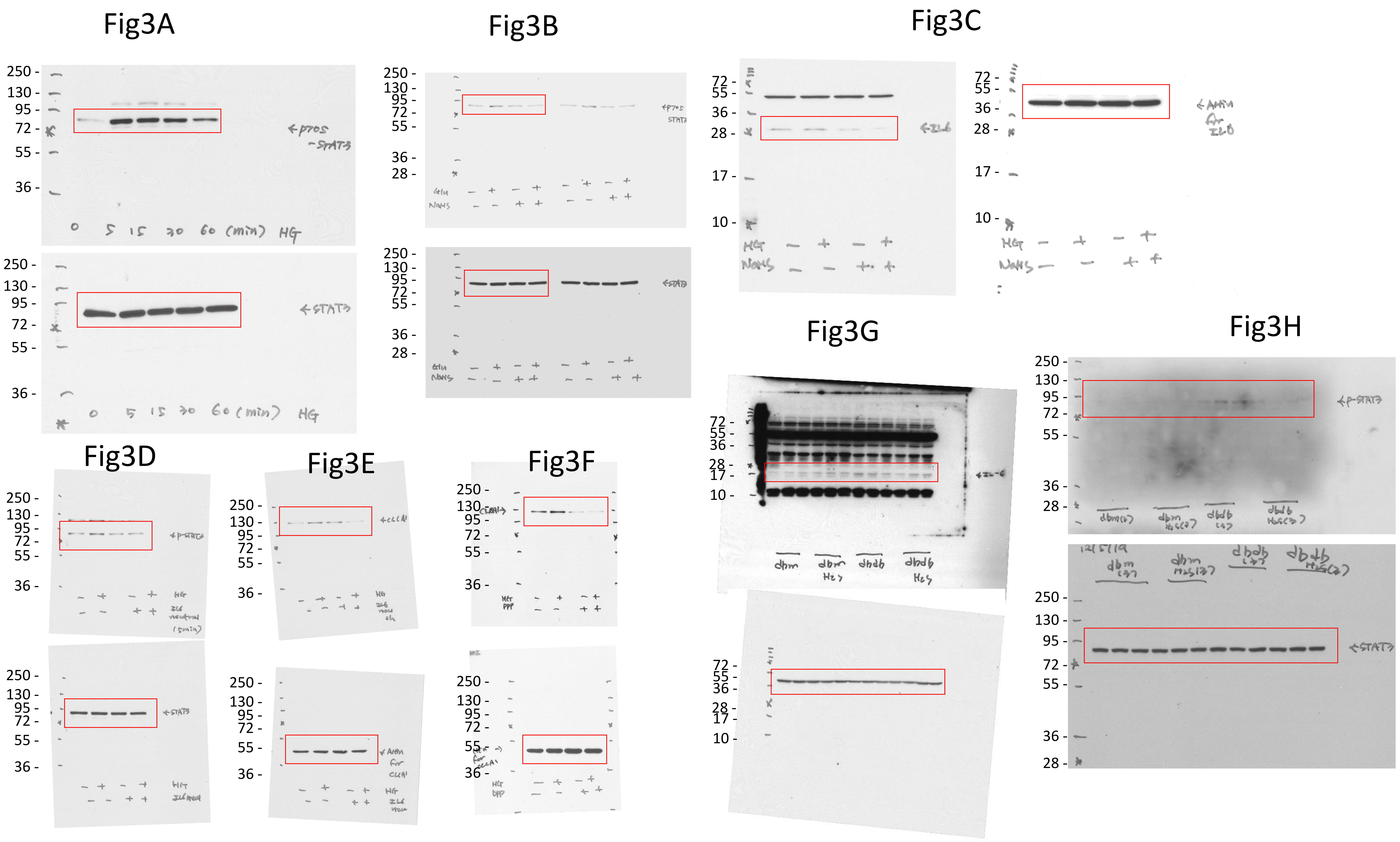
Fig3A
Fig3B
Fig3C
Fig3D
Fig3E
Fig3F
Fig3G
Fig3H

Fig4A

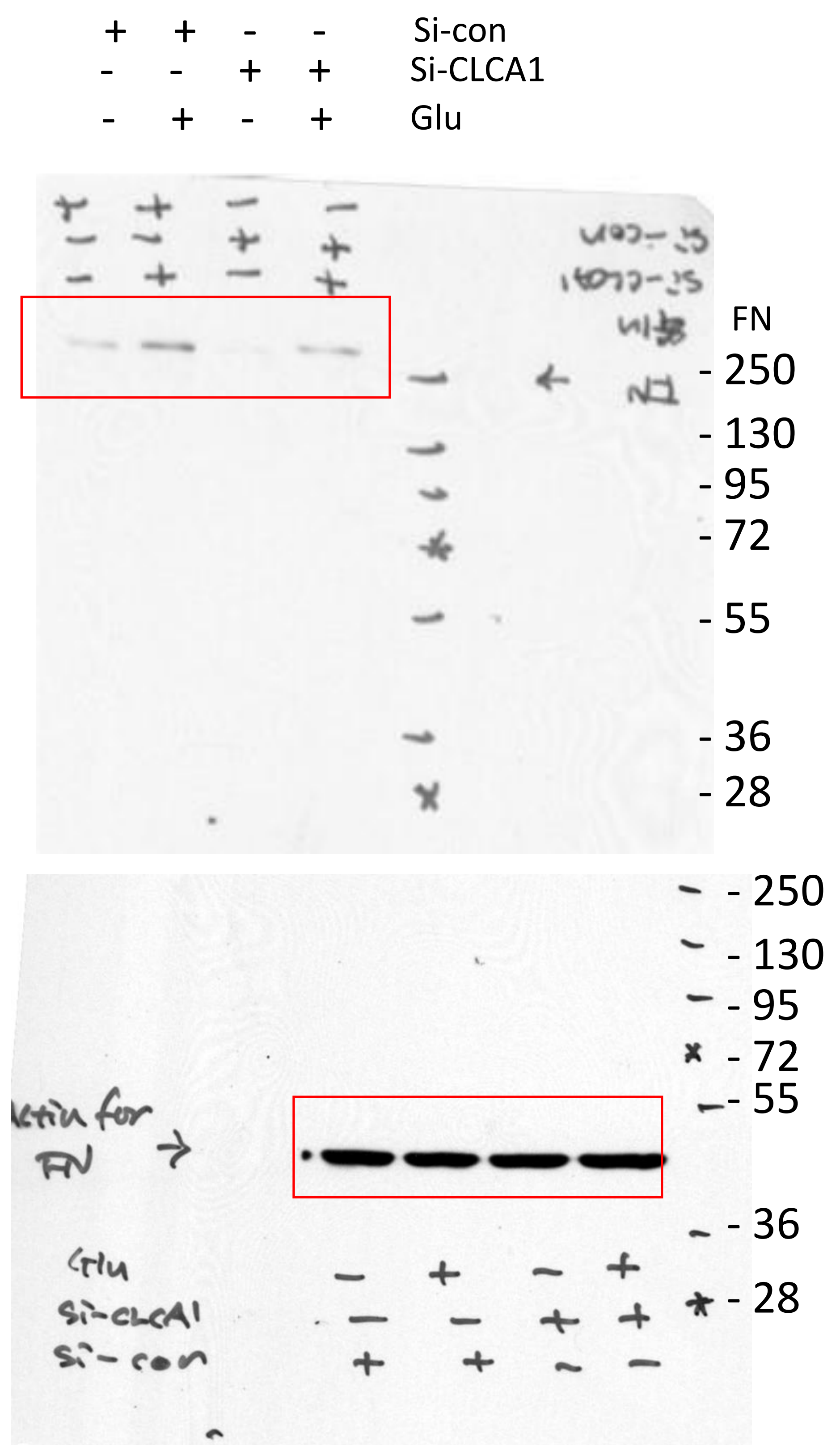

Fig4B

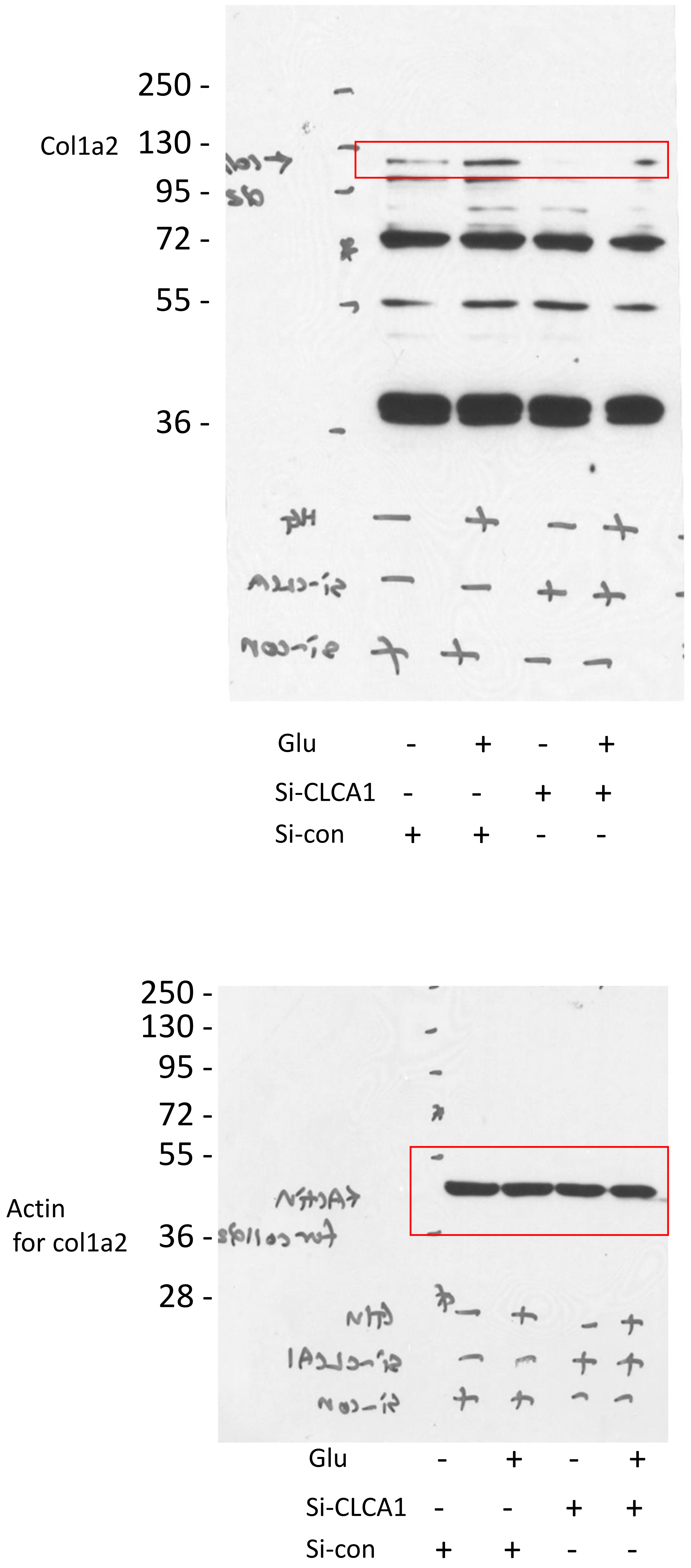

Fig4C

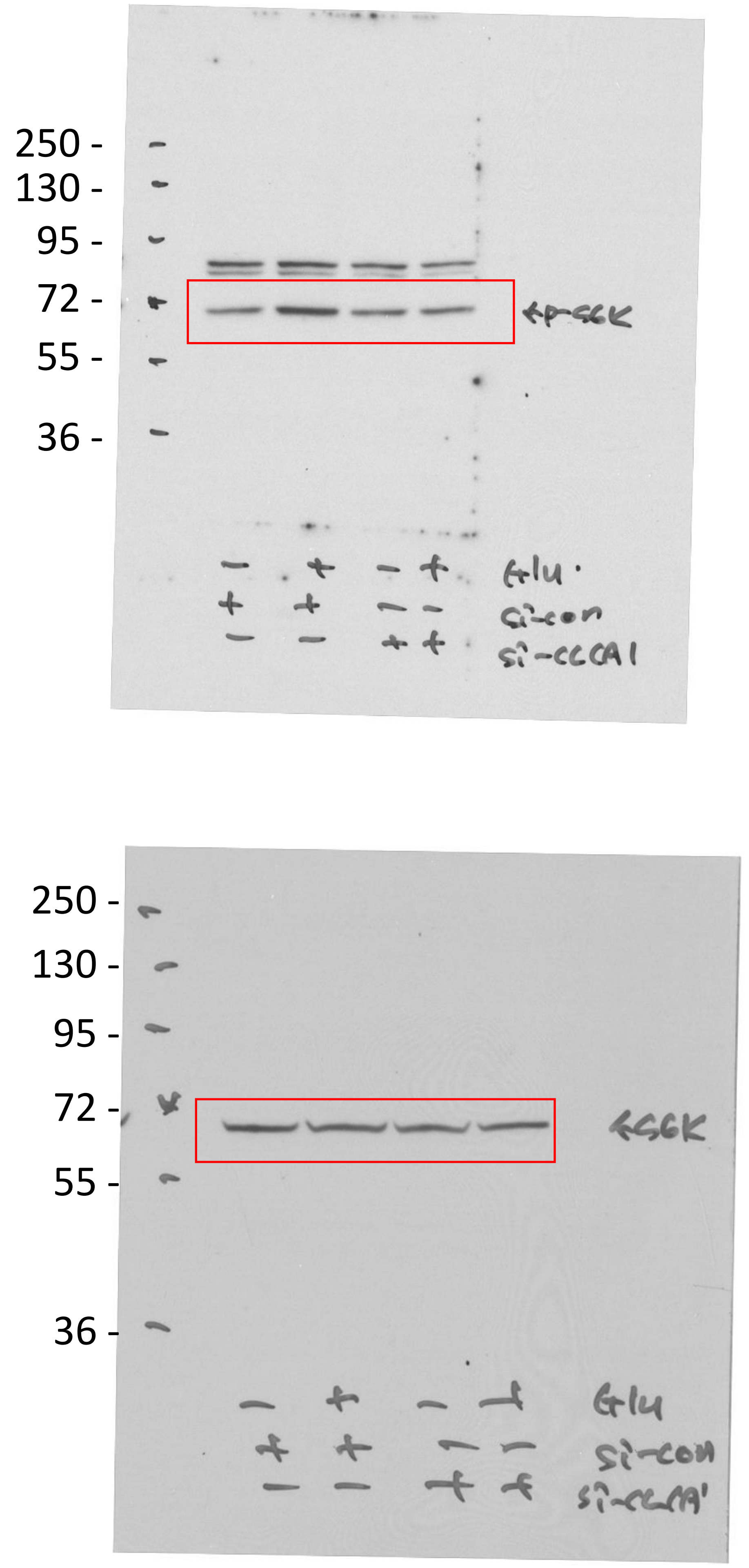

Fig4D

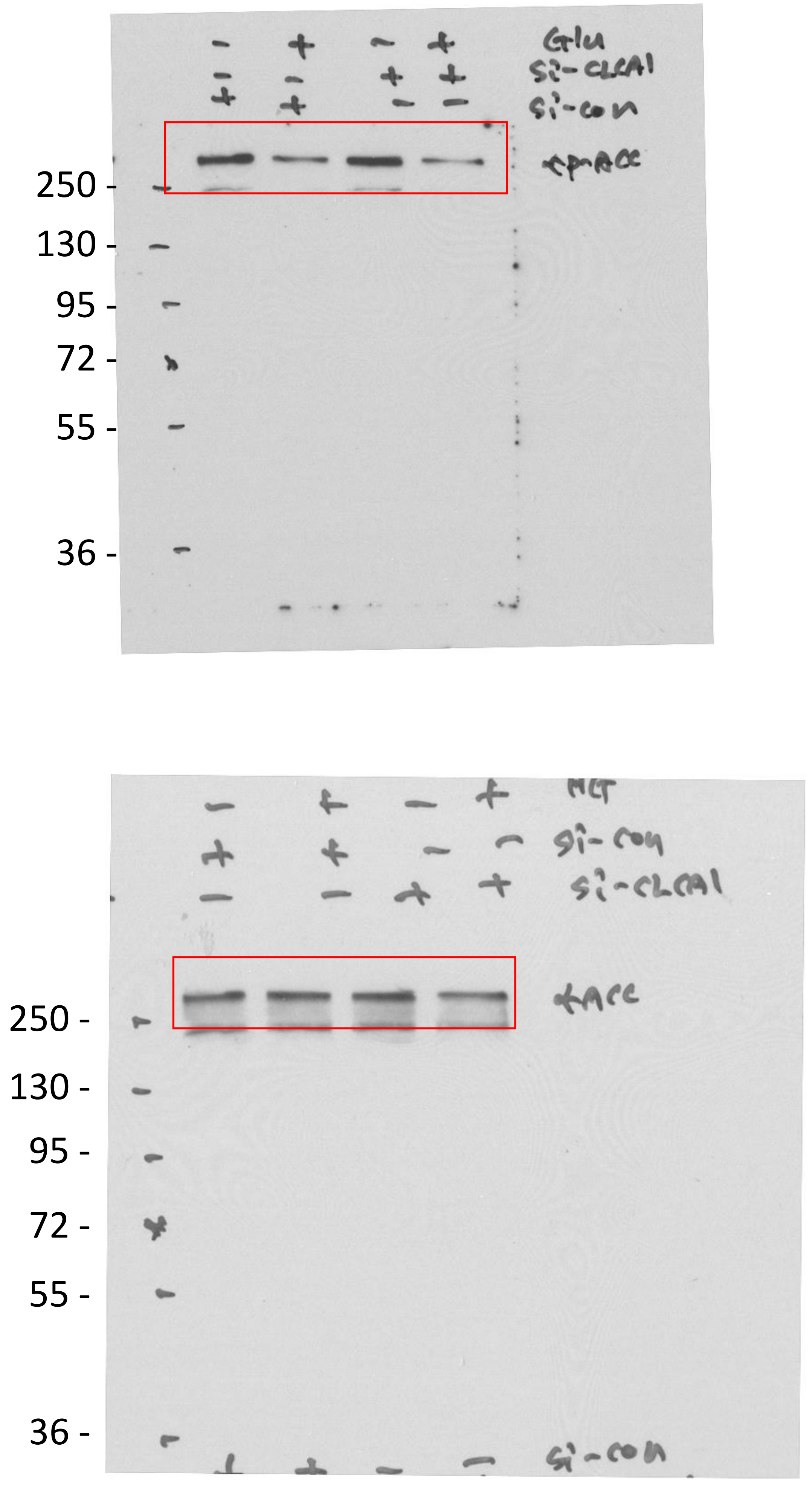

Fig5B

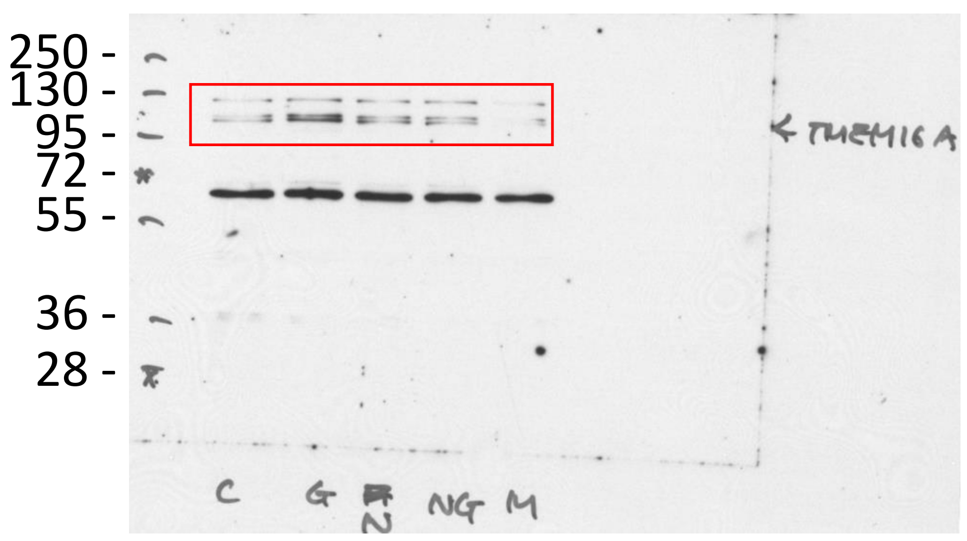
250 -
130 -
95 -
72 -
55 -
36 -
28 -
← TMEM16A
C G N NG M

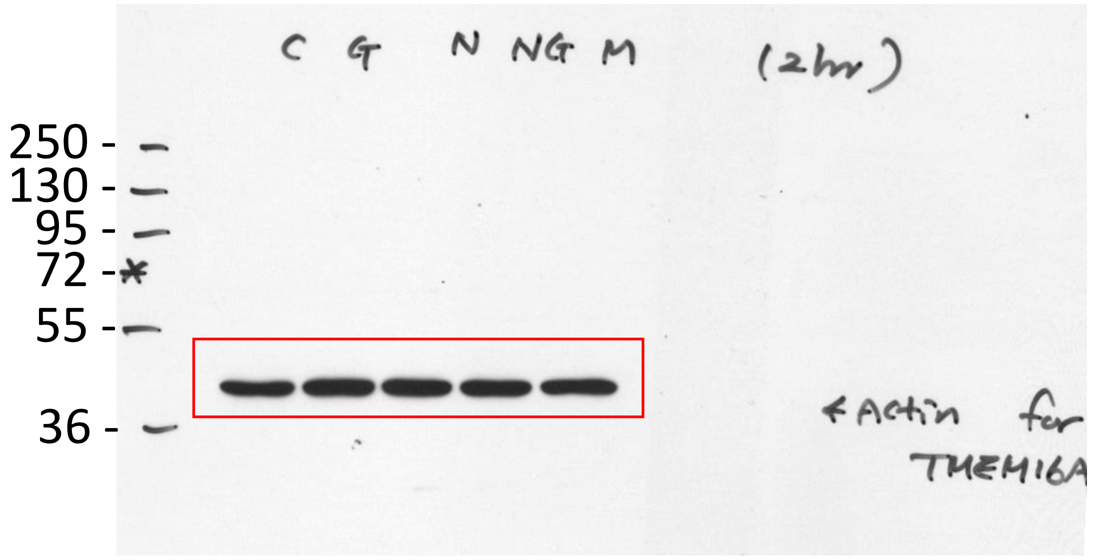
C G N NG M (2hr)
250 -
130 -
95 -
72 -
55 -
36 -
← Actin for TMEM16A

Fig5E

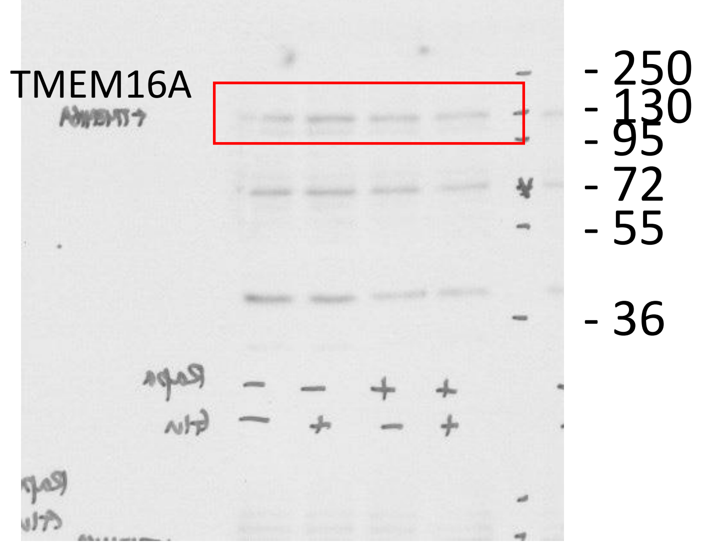
TMEM16A
- 250
- 130
- 95
- 72
- 55
- 36

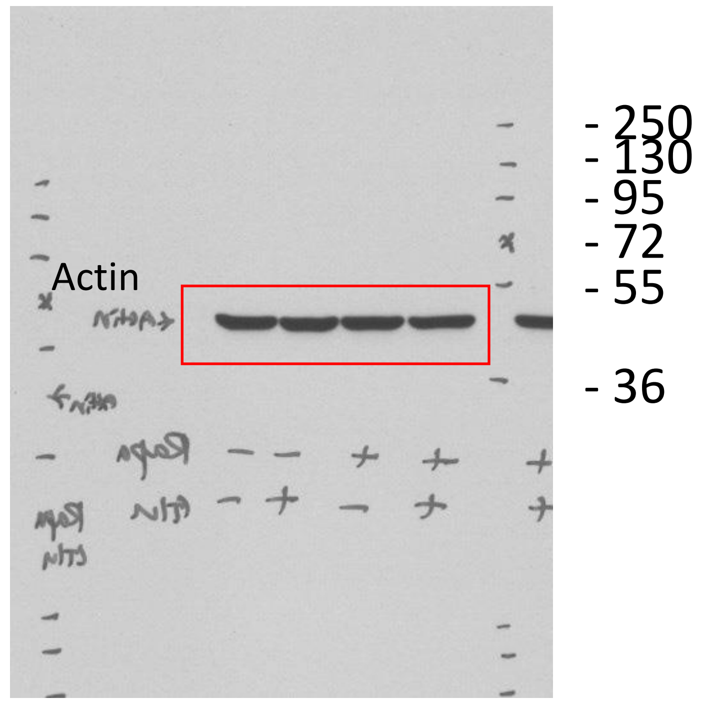
Actin
- 250
- 130
- 95
- 72
- 55
- 36

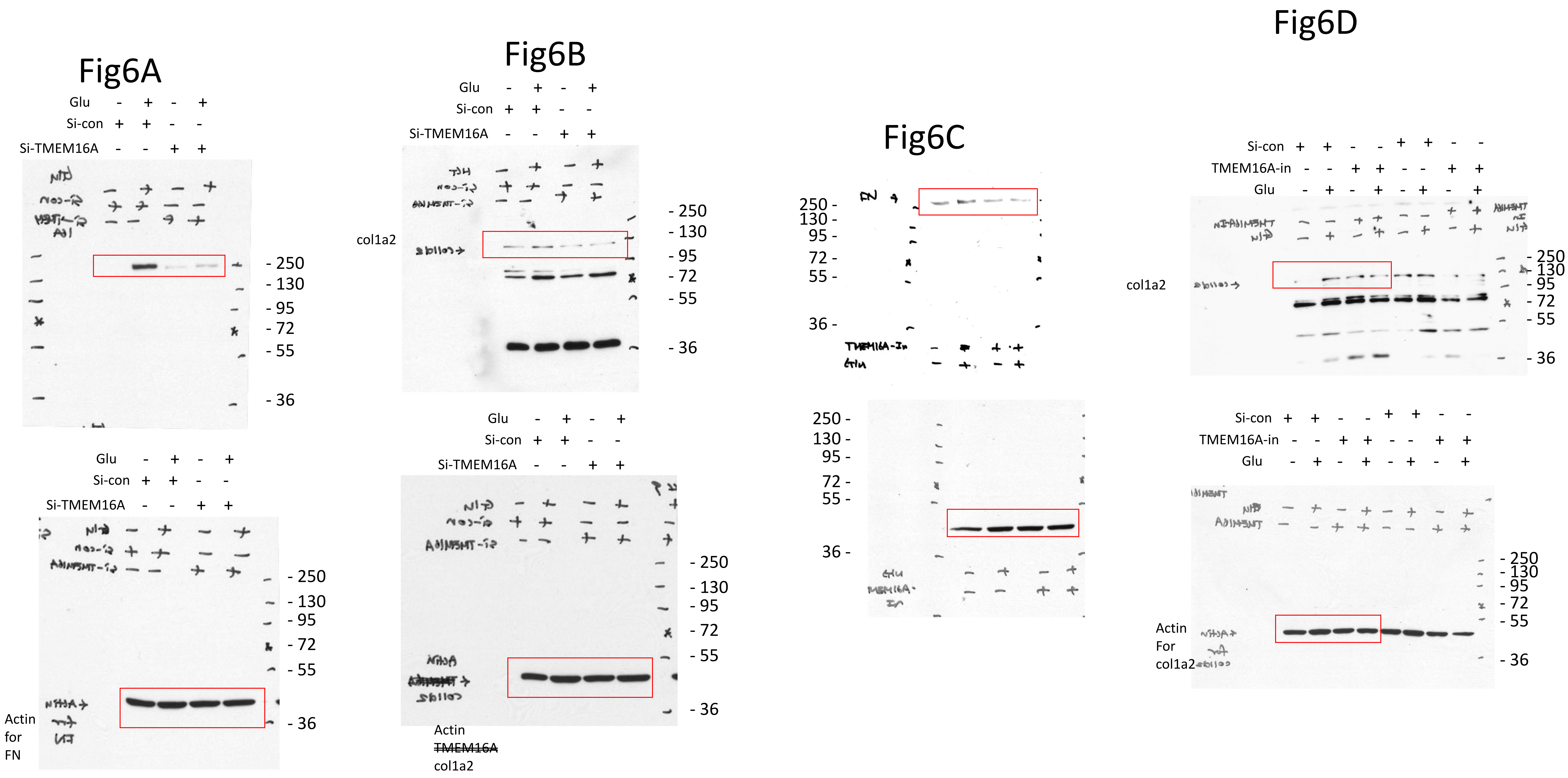
Fig6A
Glu - + - +
Si-con + + - -
Si-TMEM16A - - + +
- 250
- 130
- 95
- 72
- 55
- 36
Glu - + - +
Si-con + + - -
Si-TMEM16A - - + +
- 250
- 130
- 95
- 72
- 55
- 36
Actin for FN
Fig6B
Glu - + - +
Si-con + + - -
Si-TMEM16A - - + +
col1a2
- 250
- 130
- 95
- 72
- 55
- 36
Glu - + - +
Si-con + + - -
Si-TMEM16A - - + +
- 250
- 130
- 95
- 72
- 55
- 36
Actin
TMEM16A
col1a2
Fig6C
250 -
130 -
95 -
72 -
55 -
36 -
250 -
130 -
95 -
72 -
55 -
36 -
Fig6D
Si-con + + - - + + - -
TMEM16A-in - - + + - - + +
Glu - + - + - + - +
col1a2
- 250
- 130
- 95
- 72
- 55
- 36
Si-con + + - - + + - -
TMEM16A-in - - + + - - + +
Glu - + - + - + - +
- 250
- 130
- 95
- 72
- 55
- 36
Actin For col1a2

Fig6E

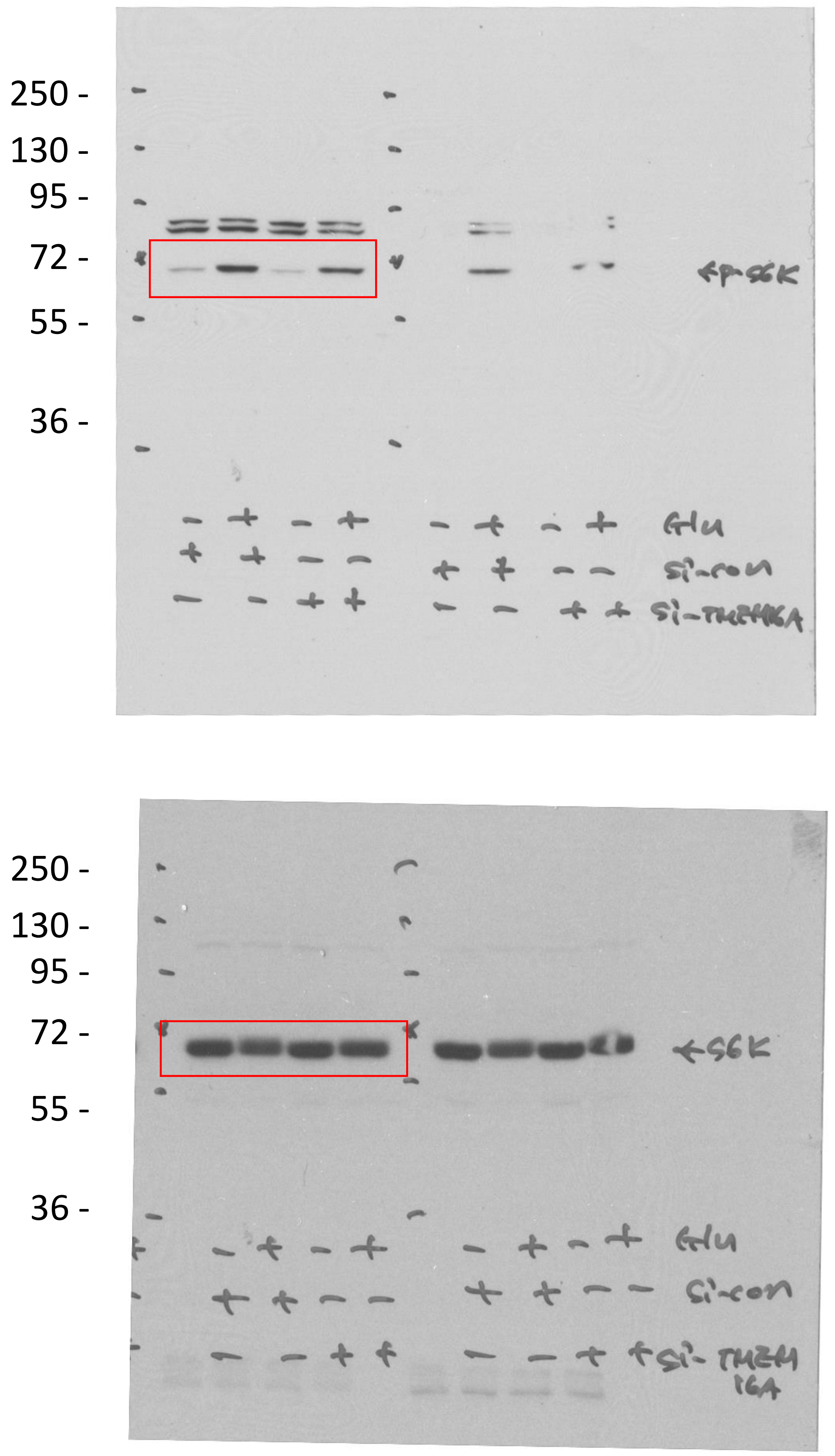
250 -
130 -
95 -
72 -
55 -
36 -
250 -
130 -
95 -
72 -
55 -
36 -

Fig6F

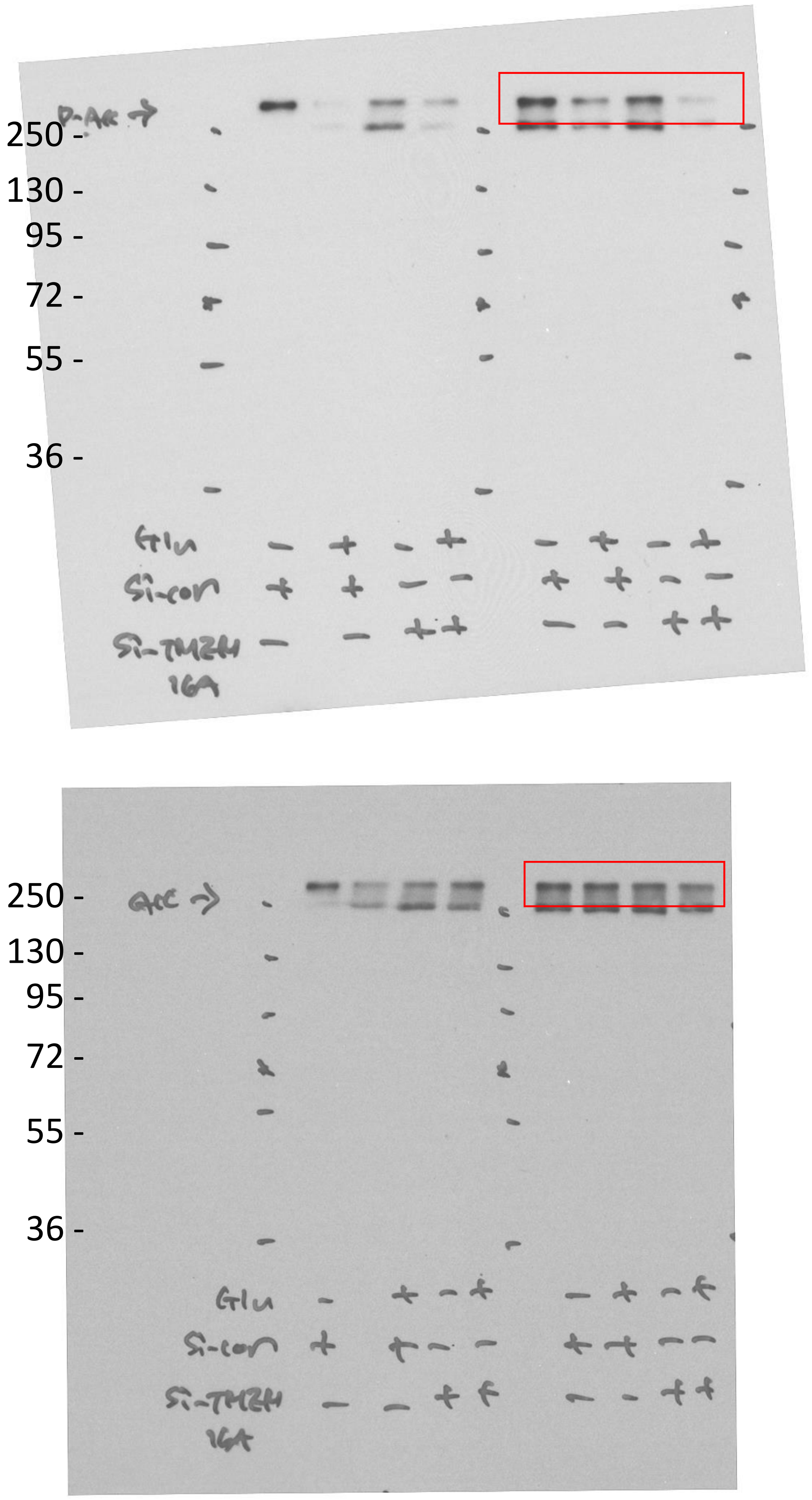
250 -
130 -
95 -
72 -
55 -
36 -
250 -
130 -
95 -
72 -
55 -
36 -

Fig. S2

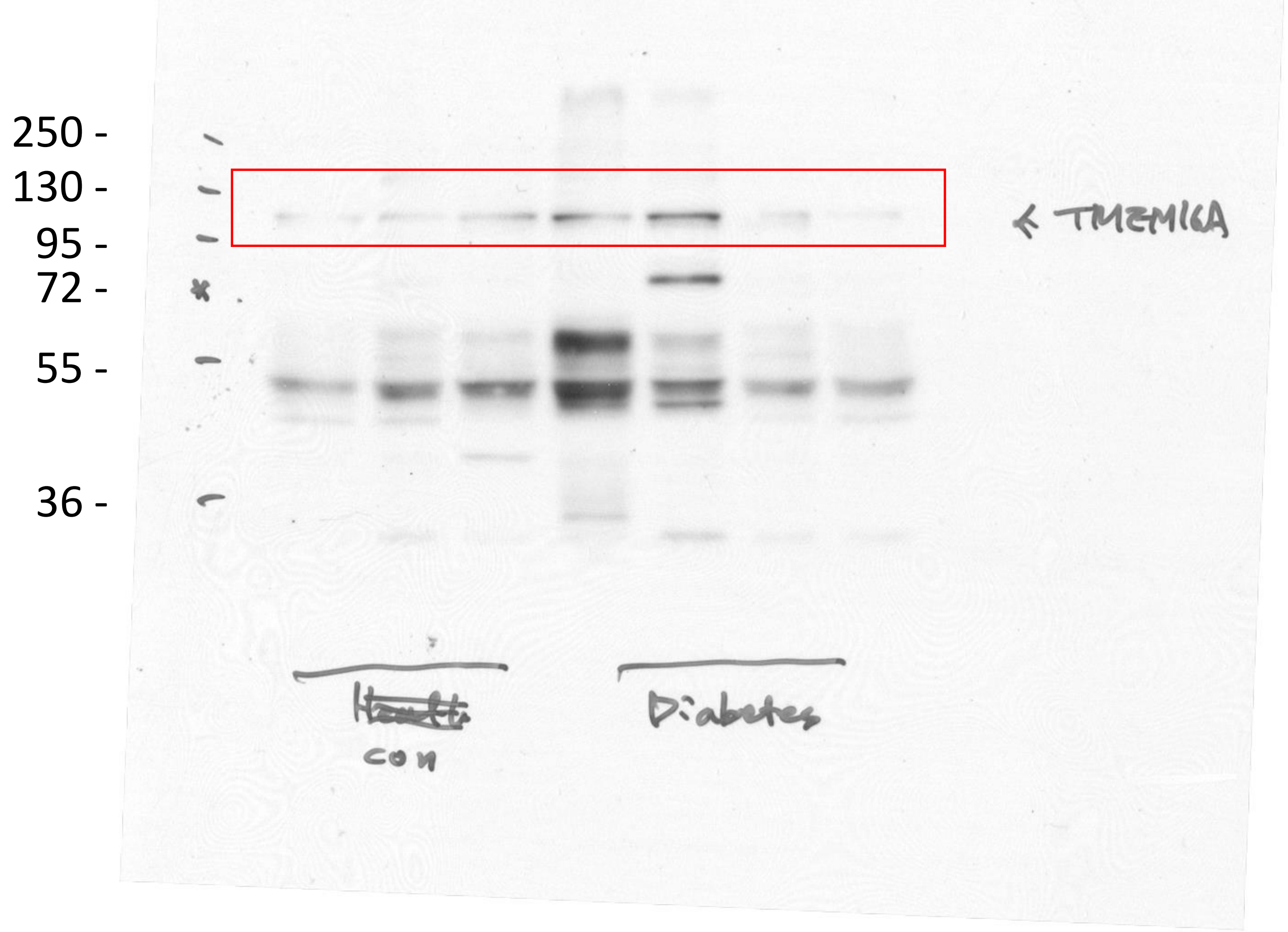

250 -
130 -
95 -
72 -
55 -
36 -
← TMEM16A
Health
con
Diabetes

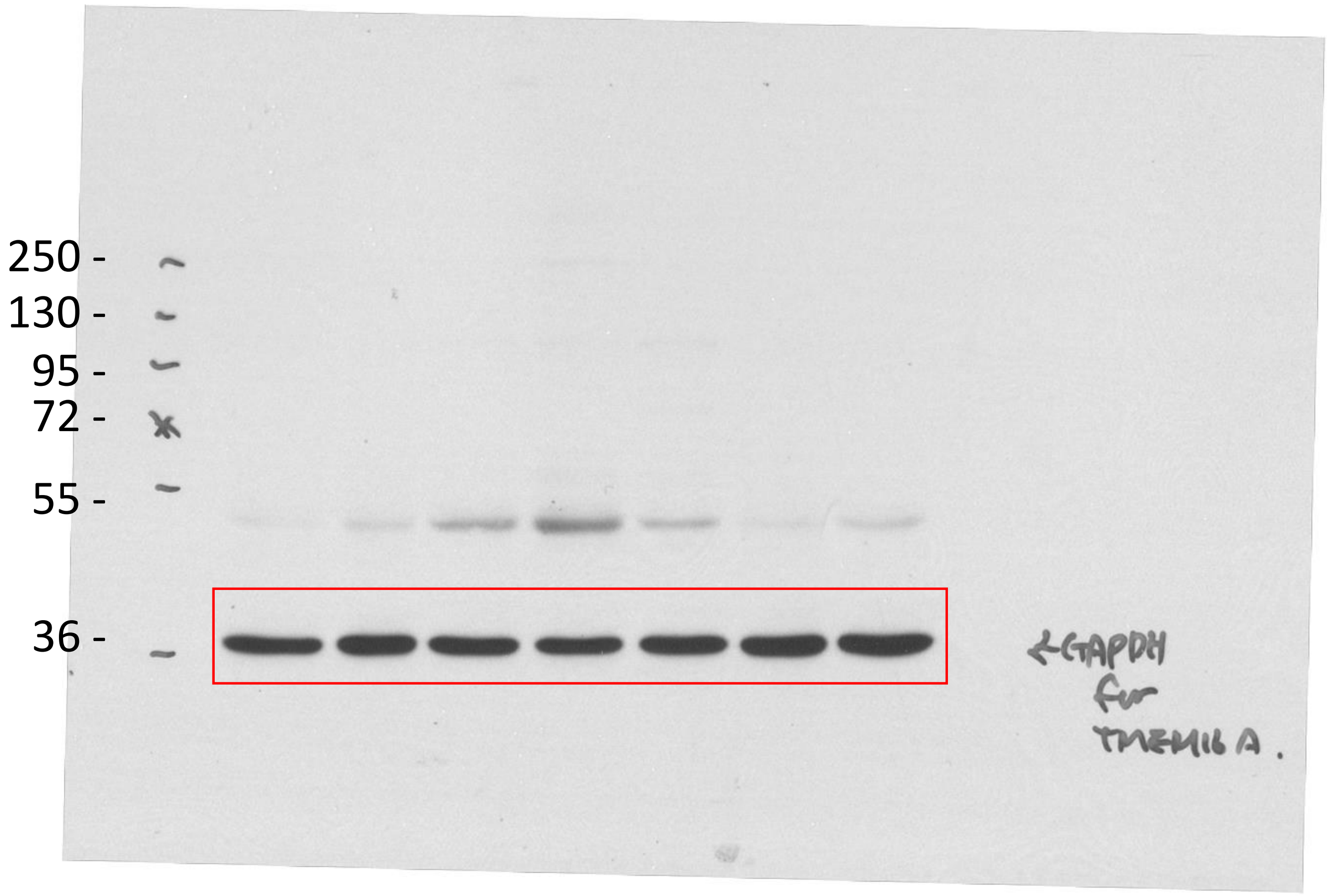

250 -
130 -
95 -
72 -
55 -
36 -
←GAPDH for TMEM16A.

Fig. S3A

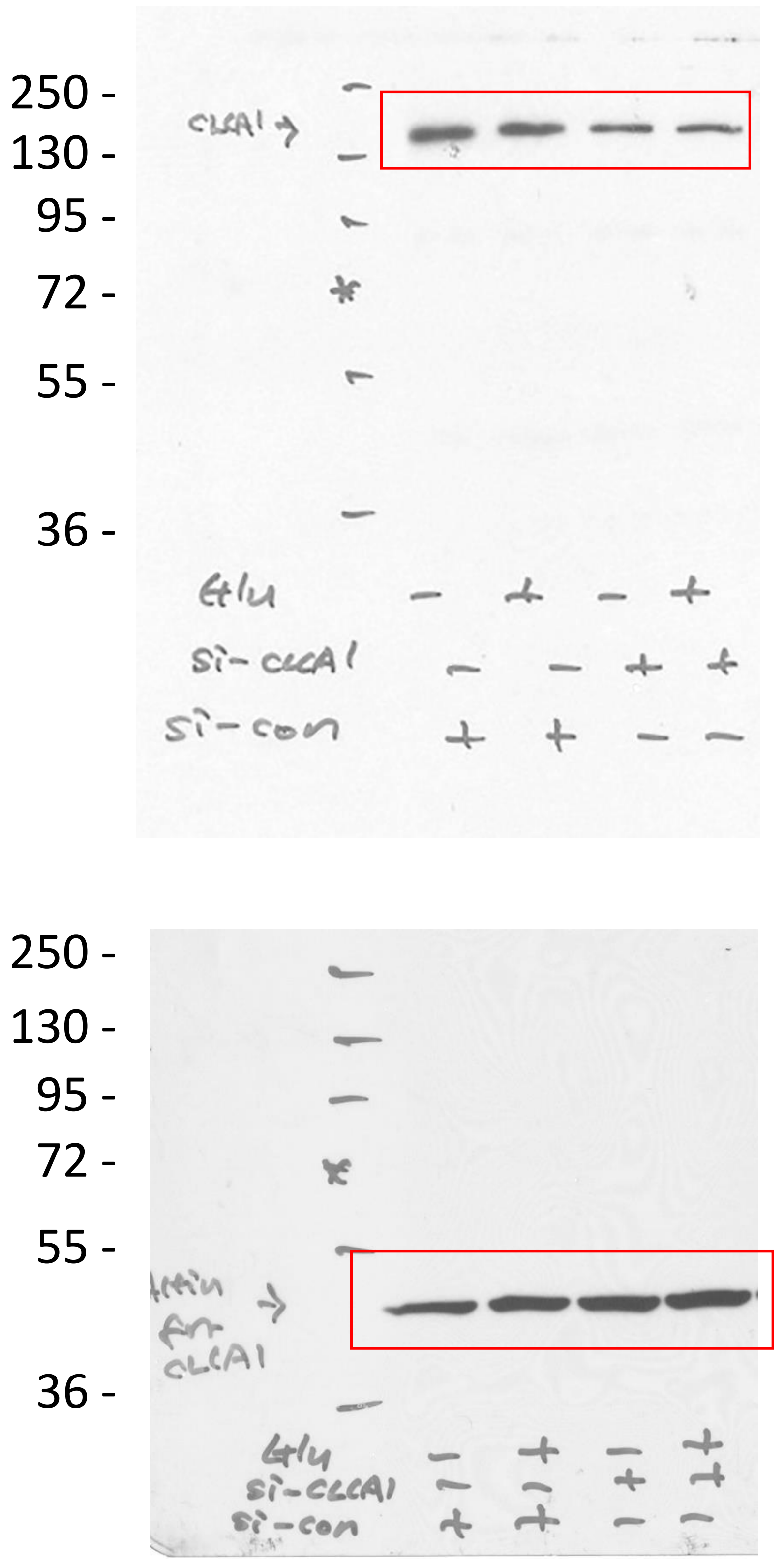

Fig. S3B

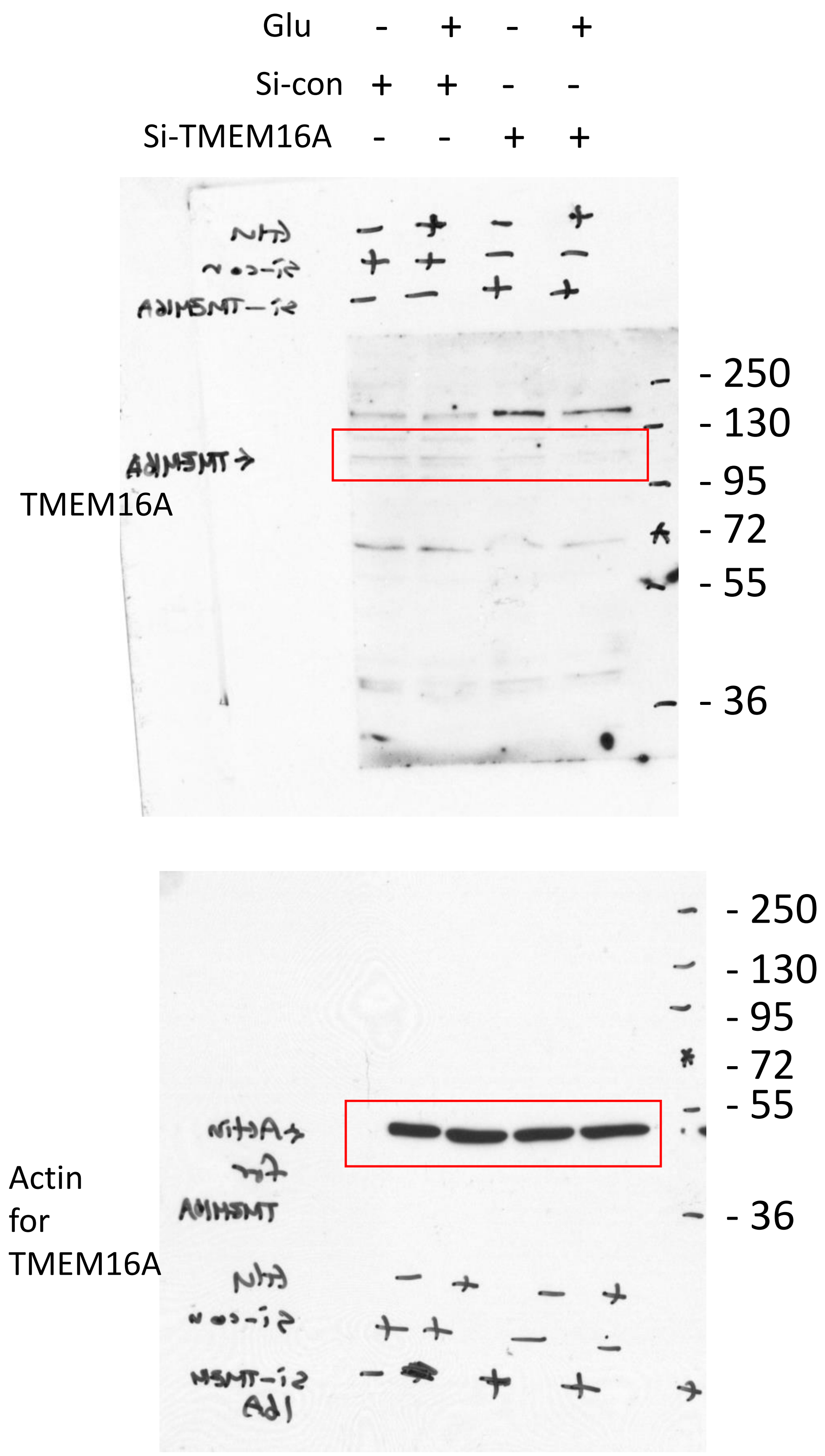

Fig. S4A

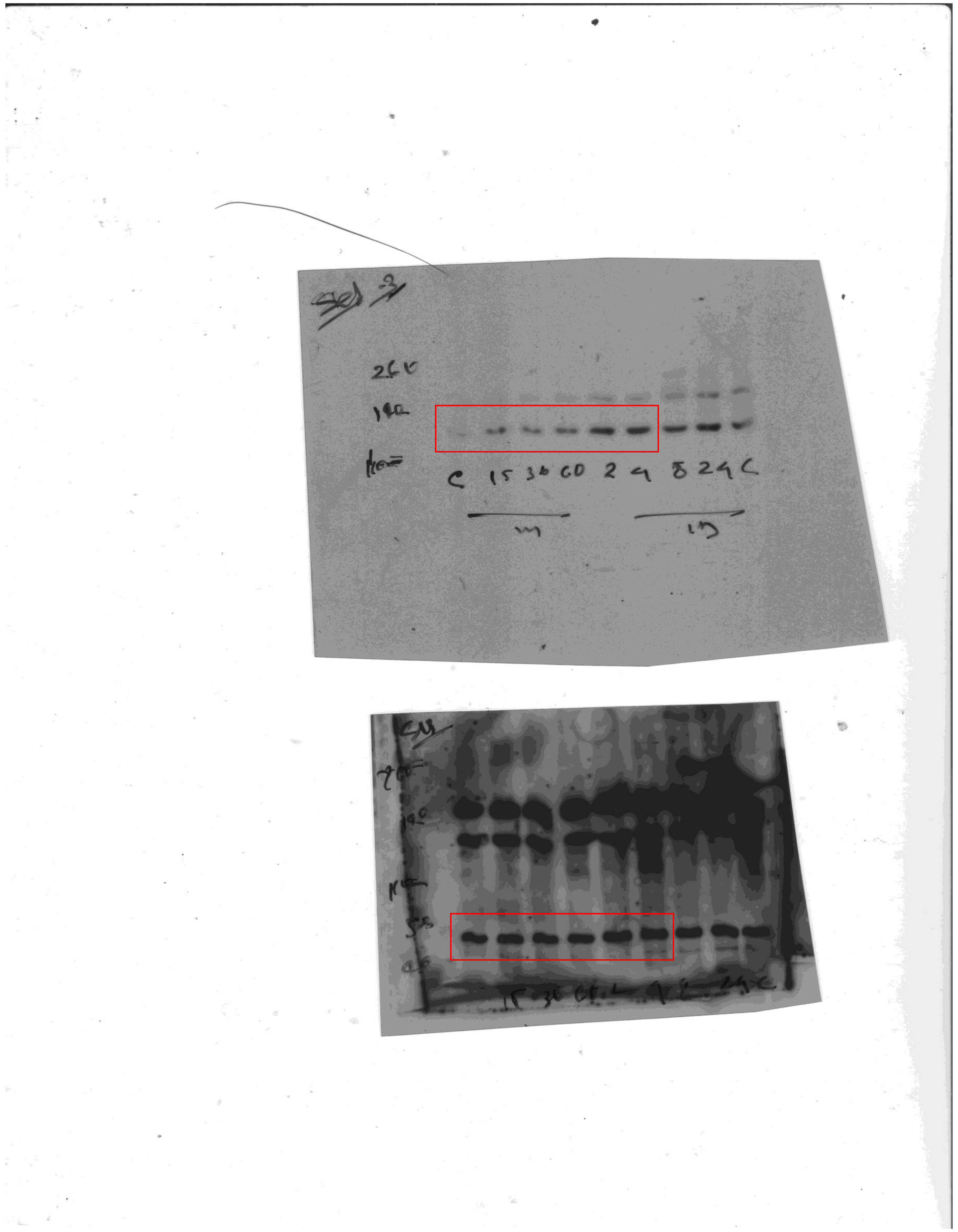

Supplement: Unedited blot and gel images [file jciinsight-10-174848-s011.pdf]
